# Placebo‐Referenced Class‐Level Treatment Effects on Chronic Kidney Disease Progression in Patients With Diabetes: A Network Meta‐Analysis

**DOI:** 10.1002/edm2.70285

**Published:** 2026-07-16

**Authors:** Ravi Kumar Pandey, Maryam Imran, Ishba Manal, Muhammad Ahmad, Aliya Noor, Ali Rohan

**Affiliations:** ^1^ Nepalgunj Medical College Nepalgunj Nepal; ^2^ Fatima Jinnah Medical University Lahore Pakistan; ^3^ Dow University of Health Sciences Karachi Pakistan; ^4^ Sharif Medical City Lahore Pakistan; ^5^ King Edward Medical University Lahore Pakistan; ^6^ The University of Lahore, University College of Medicine and Dentistry Lahore Pakistan

**Keywords:** chronic kidney disease, chronic kidney disease progression, diabetes mellitus, diabetic kidney disease, end‐stage kidney disease, network meta‐analysis, sodium‐glucose cotransporter‐2 inhibitors

## Abstract

**Background and Aim:**

Multiple pharmacologic therapies reduce chronic kidney disease (CKD) progression in patients with diabetes; however, the absence of head‐to‐head trials and heterogeneity across studies limits the interpretation of indirect comparisons. We evaluated class‐level treatment effects within a predominantly placebo‐centred evidence network.

**Methods:**

We conducted a systematic review and class‐level frequentist random‐effects network meta‐analysis of randomised controlled trials evaluating major pharmacologic therapy classes for CKD progression in patients with diabetes. The primary outcome was CKD progression, defined according to the principal composite kidney endpoint reported in each trial. Exploratory analyses evaluated albuminuria reduction and trial‐level surrogate associations between albuminuria and clinical kidney outcomes.

**Results:**

Nine trials involving 37,749 participants were included. The evidence network was star‐shaped with placebo as the central comparator. All therapeutic classes were associated with reduced CKD progression compared with placebo. Sodium‐glucose cotransporter‐2 inhibitors (SGLT2i) demonstrated the most precise estimate (HR 0.66, 95% CI 0.60–0.74) and were supported by high‐confidence evidence. Confidence in placebo‐referenced estimates for other therapeutic classes ranged from moderate to low because of indirectness, limited trial numbers and differences in study design and patient populations. Findings were generally consistent across sensitivity analyses. No significant association was observed between treatment‐induced albuminuria reduction and clinical kidney outcomes (*R* ^2^ = 0.007), although interpretation was limited by ecological bias and the small number of included studies.

**Conclusions:**

Multiple pharmacologic classes were associated with reduced CKD progression compared with placebo in patients with diabetes. Among placebo‐referenced comparisons, the most consistent and highest‐confidence estimates were observed for SGLT2i. However, the predominantly placebo‐centred network, absence of direct active‐comparator trials and important transitivity concerns limit inference regarding comparative efficacy among therapies. Findings should therefore be interpreted as placebo‐referenced class‐level treatment effects rather than evidence of comparative superiority. Direct comparative effectiveness and combination‐therapy trials are needed to define optimal treatment strategies.

## Graphical abstract

Multiple pharmacologic therapy classes were associated with reduced chronic kidney disease progression in patients with diabetes. However, because the evidence network was predominantly placebo‐centred and lacked direct active‐comparator trials, findings should be interpreted as placebo‐referenced treatment effects rather than evidence of comparative superiority. CINeMA, confidence in network meta‐analysis; CKD, chronic kidney disease; eGFR, estimated glomerular filtration rate; ET antagonists, endothelin receptor antagonists; GLP‐1RA, glucagon‐like peptide‐1 receptor agonist; HR, hazard ratio; nsMRA, nonsteroidal mineralocorticoid receptor antagonist; RAS, renin‐angiotensin system; SGLT2i, sodium‐glucose cotransporter‐2 inhibitor.

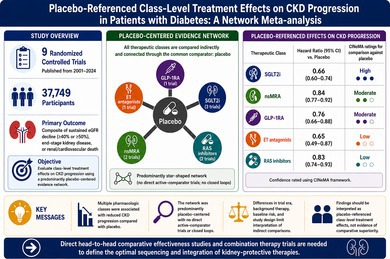

**Abbreviations**
CKD: chronic kidney disease
eGFR: estimated glomerular filtration rate
ESKD: end‐stage kidney disease
ET antagonists: endothelin receptor antagonists
GLP‐1RA: glucagon‐like peptide‐1 receptor agonist
nsMRA: nonsteroidal mineralocorticoid receptor antagonist
RAS inhibitor: renin‐angiotensin system inhibitor
SGLT2i: sodium‐glucose cotransporter‐2 inhibitor
UACR: urinary albumin‐to‐creatinine ratio

## 1 Introduction

Chronic kidney disease (CKD) remains a major global public health challenge, particularly among individuals with type 2 diabetes, in whom it represents the leading cause of end‐stage kidney disease and a major contributor to cardiovascular morbidity and mortality [1]. Despite advances in contemporary care, substantial residual renal risk persists, reflecting the complex and multifactorial pathophysiology underlying CKD progression [2, 3, 4, 5, 6].

Over the past decade, multiple pharmacologic classes have demonstrated clinically meaningful benefits on kidney outcomes in patients with CKD and diabetes, including sodium‐glucose cotransporter‐2 inhibitors (SGLT2i), nonsteroidal mineralocorticoid receptor antagonists (nsMRAs), glucagon‐like peptide‐1 receptor agonists (GLP‐1RAs) and endothelin receptor antagonists (ET antagonists) [7, 8, 9, 10, 11, 12, 13]. These therapies act through distinct and potentially complementary biological pathways [14, 15, 16, 17, 18, 19].

However, the expanding range of therapies has introduced important challenges in interpreting treatment effects across pharmacologic classes. In the absence of head‐to‐head randomised trials, comparisons between therapies are inferred indirectly from studies conducted across different treatment eras, populations and background therapies.

Several recent systematic reviews and network meta‐analyses have attempted to compare kidney outcomes across emerging therapeutic classes in patients with CKD and diabetes [20]. However, most available evidence is derived from trials conducted under substantially different clinical conditions. Differences in patient selection, enrichment strategies, baseline kidney risk, and outcome definitions may limit the interpretability of indirect comparisons and comparative treatment rankings across therapies. In addition, the extent to which reductions in surrogate markers such as albuminuria translate into clinically meaningful kidney outcomes remains uncertain across therapies.

Network meta‐analysis (NMA) provides a framework to synthesise evidence across multiple interventions using a common comparator. However, in a predominantly placebo‐centred network, indirect estimates should be interpreted cautiously. In such settings, NMA may be more appropriately used to characterise placebo‐referenced class‐level treatment effects rather than to establish comparative superiority between therapies.

Accordingly, we conducted a class‐level network meta‐analysis of randomised controlled trials evaluating major pharmacologic therapy classes for CKD progression in patients with diabetes. A class‐level framework was selected because sparse network connectivity and limited direct comparisons restricted robust agent‐level estimation. Unlike prior NMAs that primarily emphasised comparative treatment rankings, our approach explicitly considered the implications of network geometry and transitivity when interpreting treatment effects. Rather than ranking competing therapies, the principal aim of this study was to characterise placebo‐referenced treatment effects while critically evaluating the assumptions required for indirect comparisons. The inclusion of FLOW provides the first dedicated kidney outcome trial of a GLP‐1 receptor agonist, substantially strengthening the evidence base for this therapeutic class.

## 2 Methods

### 2.1 Study Design and Reporting Standards

This systematic review and network meta‐analysis (NMA) was conducted and reported in accordance with the Preferred Reporting Items for Systematic Reviews and Meta‐Analyses extension for Network Meta‐Analyses (PRISMA‐NMA) [21].

### 2.2 Data Sources, Study Selection, and Data Extraction

A comprehensive systematic search of PubMed, Embase, and Cochrane CENTRAL was performed from inception through April 2026. The full search strategy is provided in the Supporting Appendix. The Prisma flow diagram is shown in Figure S1.

Three authors independently conducted the literature search and screening process. Retrieved records were imported into EndNote for deduplication. Titles and abstracts were screened for eligibility, followed by a full‐text review of potentially relevant studies. Disagreements were resolved through consensus, with arbitration by a fourth author when necessary.

Eligible studies were randomised controlled trials (RCTs) evaluating pharmacologic interventions for chronic kidney disease (CKD) progression in patients with diabetes.

For trials enrolling mixed diabetic and non‐diabetic populations (DAPA‐CKD and EMPA‐KIDNEY), treatment effects were extracted exclusively from prespecified diabetic subgroup estimates reported by the original trial investigators, without additional calculations or reconstruction of effect estimates. Diabetes status was defined according to the criteria used in the individual trial reports and was broadly comparable across studies. Interaction *p*‐values were not consistently reported across studies, precluding formal assessment of treatment‐effect modification by diabetes status.

Data extraction was independently performed by three authors and cross‐verified by a fourth author. Extracted variables included study characteristics, participant demographics, baseline kidney disease severity, background therapies, outcome definitions, follow‐up duration and treatment effect estimates.

### 2.3 Risk of Bias Assessment

Risk of bias for each included trial was assessed using the Cochrane Risk of Bias 2 (RoB 2) tool. Two authors independently evaluated each domain. Discrepancies were resolved through discussion with a third author.

### 2.4 Assessment of Small‐Study Effects and Publication Bias

Publication bias could not be reliably assessed because fewer than ten studies were available. Funnel plots were therefore considered exploratory, and publication bias cannot be excluded.

### 2.5 Outcome Definitions

#### 2.5.1 Primary Outcome

The primary outcome was CKD progression, defined as the principal composite kidney outcome reported in each trial. To maximise inclusion of contemporary randomised evidence while preserving clinical relevance, we extracted the primary kidney composite endpoint as defined by the original investigators. Components included ≥ 40% or ≥ 50% decline in estimated glomerular filtration rate (eGFR), doubling of serum creatinine, end‐stage kidney disease (dialysis, kidney transplantation or sustained kidney failure) and renal or cardiovascular death, according to individual trial definitions (Table S1).

Although individual trials used different thresholds for kidney function decline, these endpoints represent a common biological construct of progressive CKD and have been widely adopted as validated surrogate or clinical kidney outcomes in contemporary nephrology trials. In particular, sustained declines in eGFR of ≥ 40% and ≥ 50% were endorsed by international consensus initiatives and regulatory agencies as acceptable surrogate endpoints for CKD progression because they strongly predict progression to kidney failure while allowing more efficient clinical trial design. Doubling of serum creatinine represents an approximately 57% decline in eGFR and has historically served as a regulatory endpoint in landmark nephrology trials. End‐stage kidney disease and renal death represent definitive clinical manifestations of disease progression [22, 23].

Although, the precise composite definitions varied across trials, they were considered sufficiently similar to reflect progression along the same disease continuum. Nevertheless, because inclusion of cardiovascular death and differing eGFR thresholds could contribute to clinical heterogeneity, we performed prespecified sensitivity analyses using renal‐specific composite outcomes to evaluate the robustness of the primary findings.

#### 2.5.2 Secondary Outcomes

Secondary outcomes included:

- ≥ 50% decline in eGFR.
- ≥ 40% decline in eGFR.
- Renal‐specific composite outcomes in patients with CKD and Diabetes: These outcomes were evaluated as a secondary endpoint because renal‐specific composite outcome estimates for patients with diabetes were not consistently available as primary endpoints and were reported only in secondary or subgroup analyses in several studies.

#### 2.5.3 Exploratory Outcomes

- Change in urine albumin‐to‐creatinine ratio (UACR), evaluated as a surrogate biomarker endpoint.
- Trial‐level surrogate associations between treatment‐induced albuminuria reduction and clinical kidney outcomes.

### 2.6 Statistical Analysis

#### 2.6.1 Treatment Classification

Treatment interventions were categorised into pre‐specified therapeutic classes: sodium‐glucose cotransporter‐2 inhibitors (SGLT2i), nonsteroidal mineralocorticoid receptor antagonists (nsMRAs), glucagon‐like peptide‐1 receptor agonists (GLP‐1RAs), endothelin receptor antagonists (ET antagonists) and renin‐angiotensin system (RAS) inhibitors.

Treatment effects were modelled at the class level to facilitate network connectivity and estimate average class‐level effects. This approach assumes partial exchangeability within therapeutic classes and was intended to characterise class‐level efficacy patterns.

#### 2.6.2 Network Meta‐Analysis

A frequentist random‐effects network meta‐analysis (NMA) was performed using hazard ratios (HRs) and corresponding 95% confidence intervals as the effect measure. Random‐effects models were selected a priori because clinically and methodologically important heterogeneity across trials was anticipated, including differences in trial era, background kidney‐protective therapy, baseline kidney disease severity, enrichment strategies, follow‐up duration, and composite kidney outcome definitions. These factors were considered likely to contribute to genuine between‐study variability rather than sampling error alone. Between‐study variance was estimated using restricted maximum likelihood (REML), which has favourable statistical performance for estimating between‐study variance and is recommended for random‐effects meta‐analysis, particularly in sparse evidence networks.

Network geometry was examined to assess connectivity, identify closed loops, and evaluate the feasibility of indirect comparisons. Given the limited number of studies and the predominantly placebo‐centred network structure, treatment effect estimates were interpreted with caution.

The transitivity assumption was assessed qualitatively by comparing the distribution of clinically relevant effect modifiers across treatment classes. Prespecified effect modifiers included age, sex, body mass index (BMI), baseline estimated glomerular filtration rate (eGFR), glycated haemoglobin (HbA1c), systolic blood pressure (SBP), baseline albuminuria, background RAS inhibitors use, background SGLT2i use, trial era, follow‐up duration and the use of enrichment designs. The distribution of these prespecified effect modifiers across treatment classes is summarised in Table S2.

Global inconsistency was assessed using the design‐by‐treatment interaction model. However, because the network was predominantly star‐shaped and lacked closed loops, local inconsistency could not be formally assessed, and global inconsistency testing had limited interpretability. Statistical heterogeneity was quantified using *τ* ^2^ and the *I* ^2^ statistic.

The certainty of evidence for all comparisons was evaluated using the Confidence in Network Meta‐Analysis (CINeMA) framework, incorporating assessments of within‐study bias, reporting bias, indirectness, imprecision, heterogeneity and incoherence.

Treatment ranking metrics were deliberately omitted because the predominantly placebo‐centred network lacked direct active‐comparator evidence, making comparative rankings potentially misleading and clinically difficult to interpret.

NMA was performed only when sufficient network connectivity permitted indirect estimation. Outcomes with insufficient network connectivity were analysed using conventional pairwise random‐effects meta‐analysis.

#### 2.6.3 Pairwise Meta‐Analysis

Random‐effects pairwise meta‐analysis was conducted using the inverse‐variance method, with between‐study variance (*τ* ^2^) estimated using the REML method. Statistical heterogeneity was quantified using the *I* ^2^ statistic.

#### 2.6.4 Sensitivity Analysis

Several pre‐specified sensitivity analyses were conducted to evaluate the robustness of findings:

- Renal‐specific primary composite outcome meta‐analysis excluding cardiovascular death to assess whether inclusion of cardiovascular components influenced the primary results.
- Primary composite outcome in trials exclusively enrolling patients with CKD and diabetes to evaluate the influence of diabetic subgroup data derived from mixed‐population trials.
- Contemporary‐trial sensitivity analysis restricted to trials evaluating newer therapeutic classes (SGLT2i, GLP‐1RAs, nsMRAs and ET antagonists), excluding historical RAS inhibitor trials, to assess the influence of treatment era and evolving standards of care on treatment estimates.

#### 2.6.5 Secondary Outcomes Analysis

Class‐level NMA was performed for the outcome of ≥ 50% decline in eGFR. An NMA was also conducted using renal‐specific composite outcomes from trials exclusively enrolling patients with CKD and diabetes.

For the outcome of ≥ 40% decline in eGFR, network connectivity was insufficient for NMA; therefore, conventional pairwise random‐effects meta‐analysis was performed.

#### 2.6.6 Exploratory Outcomes Analysis

##### 2.6.6.1 Albuminuria Reduction and Surrogate Endpoint Analysis

Albuminuria reduction was assessed using the urinary albumin‐to‐creatinine ratio (UACR), which was evaluated as a surrogate biomarker endpoint. Treatment effects were extracted as ratios of geometric means as reported in the original trial publications and pooled using inverse‐variance methods. To evaluate whether treatment effects on UACR differed by pharmacologic class, trial‐level mixed‐effects meta‐regression was performed with drug class included as a categorical moderator.

A trial‐level surrogate analysis was conducted to assess the relationship between treatment effects on albuminuria reduction and clinical kidney outcomes. Weighted linear regression was performed using log‐transformed treatment effects (log hazard ratios for kidney outcomes and log ratios of UACR), with inverse‐variance weighting. The strength of association was quantified using regression coefficients and the coefficient of determination (*R* ^2^). Given the limited number of studies and use of aggregated trial‐level data, these analyses were considered exploratory and susceptible to ecological bias. Because the analysis was conducted at the trial level, it was not intended to validate albuminuria as an individual‐patient surrogate endpoint.

### 2.7 Software

All analyses were performed using R software (version 4.3.3), primarily using the netmeta and meta packages. Statistical significance was defined as a two‐sided *p*‐value < 0.05.

Detailed methodology can be found in the Supporting Appendix.

## 3 Results

### 3.1 Study and Population Characteristics

A total of nine randomised controlled trials (RCTs) comprising 37,749 participants were included in the network meta‐analysis (NMA). Participants were predominantly older adults, with mean ages ranging from 59 to 67 years and women comprised 26% to 37% of the study populations. Mean body mass index (BMI) ranged from 30 to 32 kg/m^2^. Mean baseline glycated haemoglobin (HbA1c), where reported, ranged from 7.7% to 8.5% and mean systolic blood pressure ranged from 136 to 158 mmHg. Detailed study, demographic, and clinical characteristics of the included studies are summarised in Table 1.

**TABLE 1:** Study and population characteristics.

Study	Number of participants	Mean age (years)	Female (%)	Mean BMI (kg/m^2^)	Mean baseline HbA1c (%)	Mean baseline systolic blood pressure (mmHg)
CREDENCE	4401	63	34	31	8.3	140
DAPA‐CKD*	4304	62	33	30	Not reported	137.2
EMPA‐KIDNEY*	6609	64	33	30	Not reported	136.4
FLOW	3533	67	30	32	7.8	138.5
SONAR	2648	65	26	30	7.8	136.3
FIDELIO‐DKD	5674	66	30	31	7.7	138
FIGARO‐DKD	7352	64	31	31	7.7	136
IDNT	1715	59	34	31	8.2	158
RENAAL	1513	60	37	30	8.5	152.5

*Note:* Trials with the (*) sign enrolled the non‐diabetic population as well.

Abbreviations: BMI, body mass index; eGFR, estimated glomerular filtration rate; HbA1c, glycated haemoglobin.

### 3.2 Assessment of Transitivity

Although demographic characteristics were broadly comparable across the included trials, important differences were identified in several clinical and methodological effect modifiers relevant to the transitivity assumption.

The most notable differences were related to the trial era and background therapy. Historical RAS inhibitor trials were conducted in 2001 and primarily evaluated treatment initiation in patients not receiving background RAS inhibition or contemporary kidney‐protective therapies. In contrast, more recent trials evaluated additional therapies on top of optimised background care, including near‐universal RAS inhibitor use and, in some studies, background SGLT2 inhibitor therapy (Table S2).

Additional heterogeneity was observed in kidney disease severity and eligibility criteria. Kidney function eligibility was based on serum creatinine in the historical RAS inhibitor trials but on eGFR in contemporary studies. Baseline kidney function and albuminuria/proteinuria also varied across trials, reflecting variation in baseline kidney disease severity and risk of CKD progression. Furthermore, cardiovascular disease prevalence ranged from 21% to 50.4%, reflecting additional variability in study populations.

Methodological differences were also evident. Follow‐up duration ranged from 2.0 to 3.7 years. The SONAR trial employed a responder‐enrichment design and DAPA‐CKD and EMPA‐KIDNEY enrolled mixed CKD populations, although only the diabetic subgroup was included in the present analysis.

Taken together, these differences do not preclude evidence synthesis but may reduce the plausibility and interpretability of indirect comparisons between therapeutic classes. Accordingly, network estimates were interpreted primarily as placebo‐referenced class‐level treatment effects rather than evidence of comparative efficacy or superiority between therapeutic classes.

### 3.3 Network Geometry

**FIGURE 1:**
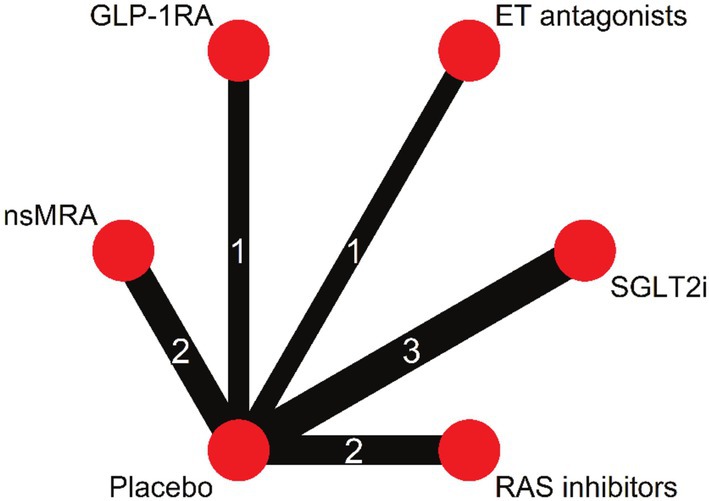
Network plot of primary composite outcome. Nodes represent treatment classes, and edges represent direct comparisons between treatments and placebo. Edge thickness is proportional to the number of trials informing each comparison. The network was predominantly placebo‐centred, with placebo serving as the common comparator linking all active therapeutic classes. No direct active‐comparator trials or closed loops were present; therefore, comparisons between active therapies were derived entirely from indirect evidence. ET antagonists, endothelin receptor antagonists; GLP‐1RA, glucagon‐like peptide‐1 receptor agonist; HR, hazard ratio; nsMRA, nonsteroidal mineralocorticoid receptor antagonist; RAS, renin‐angiotensin system; SGLT2i, sodium‐glucose cotransporter‐2 inhibitor.

The treatment network for the primary outcome included five therapeutic classes: sodium‐glucose cotransporter‐2 inhibitors (SGLT2i), nonsteroidal mineralocorticoid receptor antagonists (nsMRAs), glucagon‐like peptide‐1 receptor agonists (GLP‐1RAs), endothelin receptor antagonists (ET antagonists) and renin‐angiotensin system (RAS) inhibitors. The network was predominantly star‐shaped, with placebo serving as the central comparator linking all active treatments (Figure 1 and Table S3). The network lacked direct active‐comparator trials and contained no closed loops.

### 3.4 Heterogeneity and Inconsistency

Statistical heterogeneity was low across primary analyses, with *I* ^2^ values approximating 0%. However, given the predominantly star‐shaped network structure and the limited number of included trials, this likely reflects limited power to detect heterogeneity (Table S4). Therefore, low statistical heterogeneity should not be interpreted as evidence of clinical homogeneity.

Global assessment using the design‐by‐treatment interaction model showed no evidence of inconsistency (*Q* = 0). The absence of closed loops precluded formal assessment of local inconsistency. Consequently, the absence of statistically detected inconsistency should be interpreted cautiously.

### 3.5 Certainty of Evidence (CINeMA)

Confidence in the network estimates varied across comparisons, ranging from high to very low (Table S5).

Among placebo‐referenced comparisons, confidence was rated as high for SGLT2i versus placebo, moderate for GLP‐1RAs versus placebo and nsMRA versus placebo, and low for ET antagonists versus placebo and RAS inhibitors versus placebo. Downgrading was driven primarily by concerns regarding indirectness, particularly for comparisons involving historical RAS inhibitor trials and the ET antagonist trial.

Most indirect active‐comparator comparisons were rated as low or very low certainty. Indirectness and imprecision were the principal factors contributing to downgrading. Because the predominantly placebo‐centred network lacked closed loops, incoherence could not be formally assessed for any comparison. Overall, the CINeMA assessment highlights greater certainty in placebo‐referenced estimates than in indirect comparisons between active therapeutic classes.

### 3.6 Assessment of Small‐Study Effects

Because fewer than ten studies were available, formal assessment of small‐study effects and publication bias was inherently limited. Although comparison‐adjusted funnel plots are presented for completeness (Figure S2), visual inspection alone is insufficient to exclude publication bias. Therefore, small‐study effects and publication bias cannot be ruled out.

The network was considered sufficiently connected to permit estimation of placebo‐referenced treatment effects despite the limitations described above.

### 3.7 Primary Outcome Analysis

In the analysis of principal composite kidney outcomes, nine studies and five therapy classes were included (Table 2). All therapeutic classes demonstrated favourable placebo‐referenced treatment effects; however, the certainty of evidence varied across therapeutic classes (Table S5). Also, the evidence for the GLP‐1RA and ET antagonists was derived from a single outcome trial.

**TABLE 2:** Placebo‐referenced treatment effects, evidence certainty and key limitations across therapeutic classes.

Class	Trials	Participants	HR	CINeMA ratings for comparison against placebo	Major limitations
SGLT2i	3	15,314	0.66 (0.60–0.74)	High	Mixed populations
nsMRA	2	13,026	0.84 (0.77–0.92)	Moderate	Indirectness
GLP‐1RA	1	3533	0.76 (0.66–0.88)	Moderate	Single trial
ET antagonists	1	2648	0.65 (0.49–0.87)	Low	Enrichment design
RAS inhibitors	2	3228	0.83 (0.74–0.93)	Low	Historical trials

*Note:* Hazard ratios (HRs) are reported relative to placebo for the primary composite CKD progression outcome. CINeMA confidence ratings reflect the certainty of evidence. Major limitations represent the principal factors affecting the interpretation of treatment effects and should not be interpreted as exhaustive assessments of study quality. Given the predominantly placebo‐centred network and absence of direct active‐comparator trials, these estimates should be interpreted as placebo‐referenced class‐level treatment effects rather than evidence of comparative superiority between therapeutic classes.

Abbreviations: CINeMA, confidence in network meta‐analysis; CKD, chronic kidney disease; ET antagonists, endothelin receptor antagonists; GLP‐1RA, glucagon‐like peptide‐1 receptor agonist; HR, hazard ratio; nsMRA, nonsteroidal mineralocorticoid receptor antagonist; RAS, renin‐angiotensin system; SGLT2i, sodium‐glucose cotransporter‐2 inhibitor.

**FIGURE 2:**
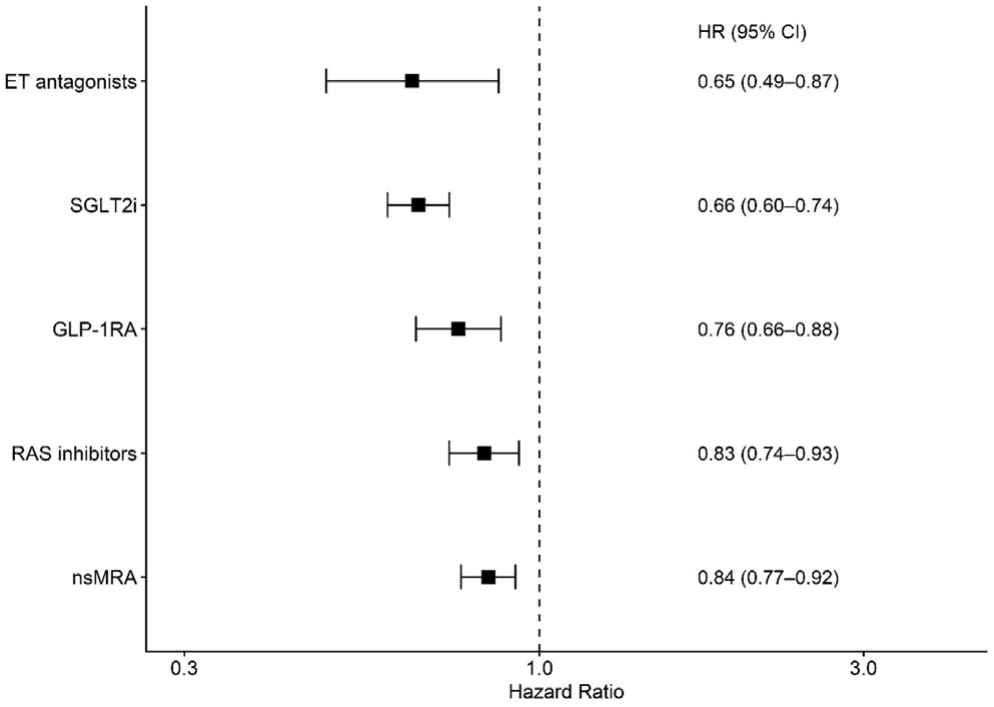
Primary composite outcome network meta‐analysis forest plot. Forest plot showing class‐level hazard ratios (HRs) for the primary composite outcome (≥ 40% or ≥ 50% decline in eGFR, end‐stage kidney disease or renal/cardiovascular death). Squares represent point estimates and horizontal lines indicate 95% confidence intervals (CIs). The vertical dashed line denotes the null effect (HR = 1).

ET antagonists (HR 0.65, 95% CI 0.49–0.87; *p* = 0.004), GLP‐1RAs (HR 0.76, 95% CI 0.66–0.88; *p* < 0.001), nsMRAs (HR 0.84, 95% CI 0.77–0.92; *p* < 0.001) and RAS inhibitors (HR 0.83, 95% CI 0.74–0.93; *p* = 0.002) were associated with a reduction in composite outcome relative to placebo (Figure 2). SGLT2i demonstrated the most precise treatment effect (HR 0.66, 95% CI 0.60–0.74; *p* < 0.001), supported by multiple trials. Given the absence of direct comparisons and differences among trials, apparent differences in effect size between therapeutic classes should not be interpreted as evidence of comparative superiority.

### 3.8 Sensitivity Analysis

#### 3.8.1 Renal‐Specific Composite Outcome Meta‐Analysis Excluding Cardiovascular Death

**FIGURE 3:**
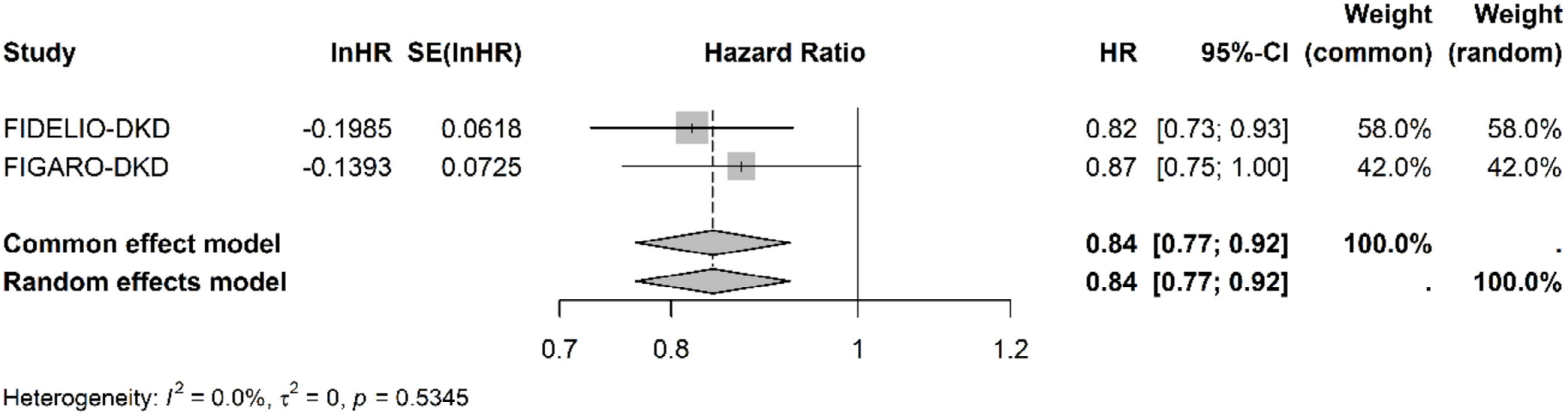
Renal‐specific composite outcome excluding cardiovascular death meta‐analysis forest plot. Forest plot of pairwise meta‐analysis evaluating nonsteroidal mineralocorticoid receptor antagonists (nsMRA) versus placebo for renal‐specific composite outcomes excluding cardiovascular death. Squares represent individual trial hazard ratios (HRs), and horizontal lines indicate 95% confidence intervals (CIs). The pooled estimate (diamond) was derived using a random‐effects model. Hazard ratios < 1 favour nsMRA therapy.

In the sensitivity analysis restricted to renal‐specific composite outcomes excluding cardiovascular death, two trials evaluating nsMRA (FIDELIO‐DKD and FIGARO‐DKD) were included. Pooled analysis demonstrated a reduction in the risk of kidney‐specific outcomes compared with placebo (HR 0.84, 95% CI 0.77–0.92), with no observed statistical heterogeneity (*I* ^2^ = 0%) (Figure 3).

For ET antagonists, the SONAR trial reported a reduction in renal‐specific composite outcomes with Atrasentan compared with placebo (HR 0.65, 95% CI 0.49–0.88). These findings were directionally consistent with the primary analysis, suggesting that the observed treatment effects were not solely driven by the inclusion of cardiovascular death within the composite outcome.

#### 3.8.2 Primary Composite Outcome in Trials Exclusively Enrolling Patients With CKD and Diabetes

Network geometry for trials enrolling patients with CKD and diabetes is presented in Figure S3. Seven studies and five therapy classes were evaluated. Compared with placebo, nsMRAs (HR 0.84, 95% CI 0.77–0.92), RAS inhibitors (HR 0.83, 95% CI 0.74–0.93) and SGLT2i (HR 0.70, 95% CI 0.59–0.83) were associated with lower risks of CKD progression (Figure S4). The similarity of these estimates to the primary analysis suggests that inclusion of diabetic subgroup data from mixed‐population trials did not materially influence the overall pattern of placebo‐referenced treatment effects.

#### 3.8.3 Sensitivity Analysis Restricted to Contemporary Trials

In this analysis, seven studies and four therapy classes were evaluated relative to a placebo. The direction and magnitude of treatment effects remained broadly consistent with the primary analysis.

Compared with placebo, nsMRAs (HR 0.84, 95% CI 0.77–0.92) and SGLT2i (HR 0.66, 95% CI 0.60–0.74) were associated with lower risks of CKD progression (Figure S5).

These findings suggest that the inclusion of older RAS inhibitor trials did not solely drive the favourable placebo‐referenced treatment effects observed in the primary analysis. However, interpretation should remain cautious because the network remained predominantly placebo‐centred and lacked direct active‐comparator trials.

### 3.9 Secondary Outcomes Analysis

#### 3.9.1 ≥ 50% Decline in eGFR (Network Meta‐Analysis)

Six studies and four therapy classes contributed to the network meta‐analysis of a ≥ 50% decline in eGFR. Compared with placebo, RAS inhibitors (HR 0.73, 95% CI 0.62–0.86) and SGLT2i (HR 0.56, 95% CI 0.48–0.66) were associated with lower risks of ≥ 50% decline in eGFR (Figure S6).

These findings were generally consistent with the primary analysis and are consistent with the placebo‐referenced kidney‐protective effects observed in the primary analysis.

#### 3.9.2 ≥ 40% Decline in eGFR (Pairwise Meta‐Analysis)

Pairwise meta‐analysis demonstrated significant reductions in the risk of ≥ 40% decline in eGFR with both SGLT2i and nsMRAs (Figure S7). No statistical heterogeneity was observed (*τ* ^2^ = 0; *I* ^2^ = 0%).

#### 3.9.3 Renal‐Specific Composite Outcomes in Patients With CKD and Diabetes (Network Meta‐Analysis)

In an analysis restricted to trials enrolling patients with CKD and diabetes and reporting renal‐specific composite outcomes, six studies and five therapeutic classes were evaluated within a predominantly placebo‐centred network.

Compared with placebo, nsMRAs (HR 0.84, 95% CI 0.77–0.92), RAS inhibitors (HR 0.79, 95% CI 0.66–0.95) and SGLT2i (HR 0.66, 95% CI 0.53–0.82) were associated with lower risks of renal‐specific composite outcomes (Figure S8). The overall pattern of treatment effects was consistent with that observed in the primary analysis.

Given the exploratory nature of this analysis, the predominantly placebo‐centred network structure and variations across trials, these findings should be interpreted cautiously.

### 3.10 Exploratory Outcome Analysis

#### 3.10.1 Albuminuria Reduction and Surrogate Endpoint Analysis

No statistically significant trial‐level association was observed between albuminuria reduction and kidney outcomes (*R* ^2^ = 0.007). Given the limited number of studies and the use of aggregated trial‐level data, these findings should not be interpreted as disproving albuminuria as a clinically relevant surrogate marker. Detailed results are provided in the Supporting Appendix A1 (Sections 3.1 and 3.2; Figures S9–S13).

### 3.11 Risk of Bias

Risk‐of‐bias assessments for individual trials are presented in the Supporting Appendix A2. Overall, most studies were judged to be at low risk of bias across assessed domains, supporting the internal validity of the included evidence.

## 4 Discussion

### 4.1 Principal Findings

In this class‐level network meta‐analysis (NMA) of randomised controlled trials (RCTs), multiple pharmacologic therapy classes were associated with a lower risk of chronic kidney disease (CKD) progression in patients with diabetes. These findings were generally consistent across secondary outcomes and prespecified sensitivity analyses, including analyses restricted to contemporary trials and trials exclusively enrolling patients with CKD and diabetes.

The certainty of placebo‐referenced treatment estimates varied across comparisons. The SGLT2i versus placebo comparison was supported by multiple large outcome trials and was rated as high confidence in the CINeMA assessment. Confidence was moderate for the placebo comparisons involving nsMRAs and GLP‐1RAs. ET antagonists and RAS inhibitors versus placebo comparisons were downgraded because of concerns regarding indirectness arising from differences in trial era, study design, background therapy, and patient populations. In addition, treatment estimates for GLP‐1RAs and ET antagonists were informed primarily by single outcome trials, contributing to greater uncertainty regarding the magnitude and generalisability of these findings.

Importantly, the predominantly placebo‐centred network, the absence of direct active‐comparator trials and identified indirectness concerns limit inference regarding comparative efficacy among therapeutic classes. Accordingly, the present analysis should be interpreted as a characterisation of placebo‐referenced class‐level treatment effects rather than evidence supporting treatment superiority or comparative ranking among therapies.

### 4.2 Interpretability of Indirect Comparisons

Interpretation of indirect comparisons in this analysis should be considered in the context of important clinical and methodological differences across trials. The most notable sources of heterogeneity included differences in treatment era, background therapy, baseline kidney risk, patient selection, and study design. Furthermore, baseline albuminuria, kidney function, blood pressure and follow‐up durations varied across studies, and the SONAR trial employed a responder‐enrichment design that may limit comparability with other trials.

Although these differences do not invalidate indirect comparisons, they represent potential challenges to the transitivity assumption and may influence the magnitude and comparability of estimated treatment effects across therapeutic classes. Consistent with these considerations, CINeMA assessments identified indirectness as a major contributor to reduced confidence for several comparisons.

### 4.3 Clinical Implications and Relationship to Contemporary Guidelines

Recent clinical practice guidelines increasingly recommend a multidrug approach to the management of CKD in patients with diabetes by combining therapies with complementary mechanisms of action to reduce residual kidney and cardiovascular risk [2, 3]. Current recommendations generally support foundational treatment with RAS blockade and SGLT2 inhibitors in eligible patients, with consideration of additional therapies such as nonsteroidal mineralocorticoid receptor antagonists (nsMRAs) and glucagon‐like peptide‐1 receptor agonists (GLP‐1RAs) according to residual kidney risk, cardiovascular comorbidities and patient‐specific factors [2, 3, 4].

The present analysis is broadly consistent with these recommendations, demonstrating favourable placebo‐referenced treatment effects across multiple therapeutic classes (Table 2). However, because the available evidence is derived predominantly from placebo‐controlled trials conducted in different clinical settings and treatment eras, these findings should not be interpreted as supporting a specific treatment sequence, hierarchy or comparative superiority among therapies.

Although treatment effects were expressed as relative measures (hazard ratios), the absolute clinical benefit of kidney‐protective therapies depends on baseline kidney risk. Participants enrolled in the included trials differed substantially with respect to kidney function, albuminuria, cardiovascular comorbidity, and background therapy, resulting in heterogeneous baseline risks and placebo event rates. Consequently, similar relative treatment effects may translate into markedly different absolute clinical benefits, with greater absolute benefit expected among patients at higher risk of CKD progression. For example, placebo kidney outcome event rates varied considerably across the landmark trials included in this review, illustrating that an identical relative treatment effect would be expected to yield larger absolute risk reductions in higher‐risk populations than in lower‐risk populations.

Because baseline risks and follow‐up durations differed substantially among studies, reliable pooled estimates of absolute treatment benefit could not be derived from the available aggregate trial data. Accordingly, the present findings should be interpreted as demonstrating consistent relative kidney protection across therapeutic classes, while recognising that the magnitude of absolute benefit will depend on the underlying risk profile of the treated population.

Increasing attention has focused on combination therapy strategies targeting complementary pathophysiologic pathways involved in CKD progression. Recent reviews have highlighted the potential benefits of combining SGLT2 inhibitors, nsMRAs, GLP‐1RAs and RAS blockade to achieve incremental cardiorenal protection [4, 5]. Emerging evidence from the CONFIDENCE trial further suggests that simultaneous initiation of finerenone and empagliflozin produces greater reductions in albuminuria than either therapy alone [6].

Nevertheless, the present analysis evaluated placebo‐referenced class‐level treatment effects and was not designed to assess additive, synergistic or sequential treatment strategies. Accordingly, the findings should not be used to infer the optimal sequencing or combination of therapies, which will require dedicated comparative‐effectiveness studies and prospective combination‐therapy trials.

### 4.4 Comparison With Prior Studies

The favourable placebo‐referenced effects observed in this analysis are broadly consistent with findings from major RCTs. The benefit observed with RAS inhibitors aligns with landmark studies such as IDNT and RENAAL, which established renin‐angiotensin system (RAS) blockade as a cornerstone of kidney protection in CKD patients with diabetes [24, 25]. Similarly, the treatment effects observed with SGLT2i are concordant with findings from CREDENCE, DAPA‐CKD and EMPA‐KIDNEY, which demonstrated substantial reductions in kidney disease progression across a range of CKD populations [7, 8, 9].

The findings for nsMRAs are consistent with FIDELIO‐DKD and FIGARO‐DKD, while the findings for GLP‐1RAs align with emerging evidence from FLOW supporting a role for these agents in cardio‐renal protection [10, 11, 12]. ET antagonists demonstrated relatively large placebo‐referenced treatment effects, consistent with the SONAR trial; however, interpretation should consider the responder‐enrichment design and lower certainty of evidence associated with this comparison [13].

Although our findings are broadly consistent with previous NMAs reporting favourable kidney effects across multiple therapeutic classes, the present study differs in several important respects. First, it incorporates contemporary evidence from recent outcome trials, including FLOW, the first dedicated kidney outcome trial evaluating a GLP‐1RA. Second, rather than emphasising treatment rankings, our analysis explicitly evaluated the implications of network geometry, indirectness and transitivity for the interpretation of treatment effects. Third, confidence in the evidence was formally assessed using the CINeMA framework. Key methodological differences between the present study and previous published NMAs are summarised in Table S6. Consequently, our conclusions extend beyond the efficacy findings of individual trials by providing an integrated assessment of both treatment effects and the certainty and interpretability of indirect comparisons within a predominantly placebo‐centred evidence network.

Importantly, these findings should not be interpreted as establishing comparative superiority between therapeutic classes. Because the available evidence is derived predominantly from placebo‐controlled trials conducted in different clinical settings and treatment eras, apparent differences in effect size may reflect differences among trials rather than true differences in efficacy.

### 4.5 Mechanistic Insights

The observation that multiple therapeutic classes were associated with reduced CKD progression in patients with diabetes is biologically plausible given the multifactorial pathophysiology of CKD progression. Contemporary therapies target different but potentially complementary pathways involved in disease progression, including hemodynamic alterations, metabolic dysfunction, inflammation, and fibrosis [14, 15, 16, 17, 18, 19].

However, mechanistic inferences should be interpreted cautiously because the present analysis was not designed to evaluate biological pathways or to determine the relative contribution of specific mechanisms to observed clinical outcomes. Consequently, the findings should not be viewed as confirmation of mechanistic hypotheses but rather as clinical evidence that several pharmacologic strategies are associated with favourable kidney outcomes compared with placebo.

Taken together, these results support the concept that CKD progression may be modified through multiple therapeutic approaches. Treatment selection should continue to be guided by patient characteristics, comorbid conditions, safety considerations, and contemporary clinical practice guidelines.

### 4.6 Safety Considerations

Although the present analysis focused on kidney efficacy outcomes, treatment selection in clinical practice should also consider the safety profiles of individual therapeutic classes. SGLT2i are generally well tolerated but have been associated with an increased risk of genital mycotic infections and, rarely, diabetic ketoacidosis. Nonsteroidal mineralocorticoid receptor antagonists (nsMRAs) may increase the risk of hyperkalemia, particularly in patients with advanced CKD. GLP‐1RAs are commonly associated with gastrointestinal adverse effects, including nausea and vomiting. ET antagonists have been associated with fluid retention and edema, which may limit their use in selected patients. RAS inhibitor trials also demonstrated risks of hyperkalemia and transient declines in kidney function (Table 3).

**TABLE 3:** Major safety findings relative to placebo reported in the included landmark trials.

Trial	Drug class	Principal adverse events reported relative to placebo	Key safety message
CREDENCE	SGLT2i	Increased genital mycotic infections; rare diabetic ketoacidosis; no excess acute kidney injury	Overall favourable safety profile with expected class‐specific adverse events
DAPA‐CKD	SGLT2i	Increased genital mycotic infections; rare diabetic ketoacidosis; no excess acute kidney injury	Favourable safety profile consistent with the SGLT2i class
EMPA‐KIDNEY	SGLT2i	Increased genital mycotic infections; rare diabetic ketoacidosis; no excess acute kidney injury	Consistent with SGLT2i class
FIDELIO‐DKD	nsMRA	Increased hyperkalemia	Hyperkalemia was the principal safety concern; careful monitoring of serum potassium is recommended.
FIGARO‐DKD	nsMRA	Increased hyperkalemia	Hyperkalemia predominated; careful monitoring of serum potassium is recommended.
FLOW	GLP‐1RA	Increased gastrointestinal adverse events (nausea, vomiting, diarrhoea)	Gastrointestinal intolerance represented the principal adverse effect
SONAR	ET antagonist	Increased edema and fluid retention	Fluid retention was the principal limitation of therapy and requires careful patient selection
RENAAL	RAS inhibitor	Increased hyperkalemia and transient increases in serum creatinine	Safety profile consistent with initiation of RAS blockade; monitoring of kidney function and serum potassium is recommended
IDNT	RAS inhibitor	Increased hyperkalemia and transient increases in serum creatinine	Safety profile consistent with initiation of RAS blockade; monitoring of kidney function and serum potassium is recommended

*Note:* Major safety findings are summarised descriptively from the original placebo‐controlled trial publications. Because adverse‐event definitions, ascertainment and reporting differed across studies, this table is intended to provide qualitative clinical context rather than direct quantitative comparisons across therapeutic classes.

Because safety outcomes were not evaluated systematically in the present analysis, the findings should be interpreted alongside evidence from individual randomised trials, clinical practice guidelines, and patient‐specific risk profiles.

### 4.7 Albuminuria as a Surrogate Marker for Clinical Kidney Outcomes

Reductions in albuminuria were observed across pharmacologic classes; however, trial‐level surrogate analysis did not demonstrate a statistically significant association between treatment‐induced albuminuria reduction and clinical kidney outcomes. These findings should be interpreted cautiously and should not be considered evidence against the utility of albuminuria as a surrogate marker of kidney disease progression.

Importantly, surrogate validation at the trial level differs fundamentally from validation at the individual‐patient level. Trial‐level analyses evaluate whether interventions producing larger average reductions in albuminuria also produce proportionally greater treatment effects on clinical kidney outcomes across studies, whereas patient‐level analyses assess whether individuals who experience greater reductions in albuminuria subsequently have lower risks of kidney disease progression. These represent distinct scientific questions and should not be interpreted interchangeably.

The present analysis was based on a small number of heterogeneous trials evaluating therapies with different mechanisms of action, baseline kidney risk, background therapies, and outcome definitions. Consequently, the observed absence of a trial‐level association (*R* ^2^ = 0.007) may reflect limited statistical power, between‐trial heterogeneity, and ecological bias rather than the absence of a biological relationship. Ecological analyses use aggregated study‐level data and cannot account for patient‐level associations, making them susceptible to ecological fallacy.

Accordingly, our findings do not invalidate albuminuria as a clinically relevant surrogate endpoint. Rather, they indicate that the available trial‐level evidence is insufficient to demonstrate a consistent quantitative relationship between the magnitude of albuminuria reduction and treatment effects on clinical kidney outcomes across diverse therapeutic classes. This interpretation remains consistent with individual patient‐level meta‐analyses demonstrating that reductions in albuminuria are associated with lower subsequent risks of CKD progression and kidney failure [26, 27, 28, 29, 30, 31].

### 4.8 Limitations

Several limitations should be acknowledged. First, the evidence network was predominantly placebo‐centred and lacked direct active‐comparator trials or closed loops. Consequently, local inconsistency could not be formally assessed and all comparisons between active therapies relied on indirect evidence. This represents an important limitation of the present analysis and restricts the ability to draw robust conclusions regarding comparative efficacy between therapeutic classes. Accordingly, the findings are best interpreted as placebo‐referenced class‐level treatment effects rather than evidence of comparative superiority among therapies.

Second, important differences existed across trials with respect to treatment era, background therapy, baseline kidney risk, follow‐up duration, and study design. Earlier RAS inhibitor trials evaluated treatment initiation in the absence of contemporary kidney‐protective therapies, whereas more recent studies assessed additional therapies on top of optimised background care. Furthermore, variations in albuminuria, kidney function, blood pressure, and the responder‐enrichment design used in the SONAR trial may have challenged the transitivity assumption and influenced the interpretability of indirect comparisons.

Additional stratified analyses according to baseline eGFR, albuminuria and background use of contemporary therapies were not feasible because of the limited number of studies within individual treatment classes and reliance on aggregate trial‐level data.

Third, in DAPA‐CKD and EMPA‐KIDNEY, effect estimates for patients with diabetes were obtained from prespecified subgroup analyses rather than trials exclusively enrolling diabetic populations. Although diabetes subgroup definitions were broadly comparable across studies, subgroup‐derived estimates may be less precise than estimates obtained from dedicated CKD trials done only in diabetics.

Fourth, this was a class‐level network meta‐analysis, and therefore the results represent average treatment effects within pharmacologic classes rather than individual agents. Potential differences among specific drugs within a therapeutic class could not be evaluated. Because baseline risks and follow‐up durations varied considerably across studies, absolute treatment benefits could not be estimated reliably from the available trial‐level data.

Fifth, analyses were based on trial‐level aggregate data rather than individual patient‐level data. Consequently, treatment‐effect modification according to age, sex, baseline kidney function, albuminuria, cardiovascular disease status or background medications could not be assessed. This limits the ability to determine whether the magnitude of treatment benefit differs across clinically important patient subgroups and may reduce the applicability of findings to individualised treatment decisions.

Sixth, the primary outcome incorporated composite kidney endpoints that varied across trials, including different thresholds of eGFR decline (≥ 40% or ≥ 50%), end‐stage kidney disease and renal or cardiovascular death. These components do not represent identical clinical events and may be influenced differently by individual therapies. Although sensitivity analyses restricted to renal‐specific outcomes yielded broadly consistent findings, heterogeneity in endpoint definitions may have affected comparability across studies.

Finally, the surrogate endpoint analysis was based on a limited number of studies and used aggregated trial‐level data, making it susceptible to ecological bias. Therefore, the absence of a statistically significant association between albuminuria reduction and clinical kidney outcomes should not be interpreted as evidence against albuminuria as a clinically relevant surrogate marker.

## 5 Conclusions

Multiple pharmacologic classes were associated with a lower risk of chronic kidney disease (CKD) progression in patients with diabetes compared with placebo. However, the current evidence base is predominantly derived from placebo‐controlled trials, and differences in trial design, background therapy, and study context limit the interpretability of indirect comparisons between therapeutic classes. Accordingly, these findings should be interpreted as descriptive of placebo‐referenced class‐level efficacy patterns rather than evidence of comparative superiority. Direct comparative effectiveness studies and dedicated combination‐therapy trials are needed to define the optimal sequencing and integration of kidney‐protective therapies for patients with CKD and diabetes.

## Author Contributions

**Ravi Kumar Pandey:** conceptualization, methodology, formal analysis, writing – original draft, data curation, investigation, supervision, project administration. **Ishba Manal:** data curation, writing – review and editing, software. **Maryam Imran:** data curation, formal analysis, visualization, writing – review and editing. **Aliya Noor:** writing – original draft, visualization, validation. **Ali Rohan:** writing – review and editing, validation. **Muhammad Ahmad:** writing – review and editing, data curation, validation.

## Ethics Statement

IRB approval was not required because it is a synthesis of publicly available data.

## Conflicts of Interest

The authors declare no conflicts of interest.

## Supporting information


**Appendix A1:** Contains texts, figures, and tables supporting the main manuscript.
**Appendix A2:** Detailed risk‐of‐bias assessments for all included randomised controlled trials using the Cochrane Risk of Bias 2 (RoB 2) tool.

**Figure S1:** PRISMA flow diagram.
**Figure S2:** Comparison adjusted funnel plot.
**Figure S3:** Network plot for primary composite outcome in trials enrolling patients with CKD and diabetes.
**Figure S4:** Trials enrolling patients with CKD and diabetes forest plot: Random effects model.
**Figure S5:** Contemporary trials only forest plot: Random effects model.
**Figure S6:** ≥ 50% decline in eGFR outcome forest plot: Random effects model.
**Figure S7:** ≥ 40% decline in eGFR outcome pairwise meta‐analysis forest plot.
**Figure S8:** Exploratory renal specific composite outcomes in patients with CKD and diabetes forest plot.
**Figure S9:** Albuminuria reduction pairwise meta‐analysis forest plot.
**Figure S10:** Albuminuria reduction with SGLT2i pairwise meta‐analysis forest plot.
**Figure S11:** Albuminuria reduction with nsMRA pairwise meta‐analysis forest plot.
**Figure S12:** Trial‐level association between albuminuria reduction and kidney outcomes.
**Figure S13:** Trial‐level association between albuminuria reduction and kidney outcomes stratified by class.
**Table S1:** Composite outcome component across trials.
**Table S2:** Comparison of key effect modifiers across trials to assess the plausibility of the transitivity assumption.
**Table S3:** Network connectivity and design structure.
**Table S4:** Heterogeneity statistics and design‐specific *Q* decomposition.
**Table S5:** CINeMA confidence assessment.
**Table S6:** Comparison with prior network meta‐analysis.

## Data Availability

This meta‐analysis synthesised data available in public databases.
