# Supplementary material for: Placebo‐Referenced Class‐Level Treatment Effects on Chronic Kidney Disease Progression in Patients With Diabetes: A Network Meta‐Analysis
Source: Endocrinol Diabetes Metab. 2026 Jul 16;9(4):e70285. doi: 10.1002/edm2.70285 (PMC13376836; doi:10.1002/edm2.70285)
Supplement: Supplementary file 2 — Figure S1: PRISMA flow diagram. Figure S2: Comparison adjusted funnel plot. Figure S3: Network plot for primary composite outcome in trials enrolling patients with CKD and diabetes. Figure S4: Trials enrolling patients with CKD and diabetes forest plot: Random effects model. Figure S5: Contemporary trials only forest plot: Random effects model. Figure S6: ≥ 50% decline in eGFR outcome forest plot: Random effects model. Figure S7: ≥ 40% decline in eGFR outcome pairwise meta‐analysis forest plot. Figure S8: Exploratory renal specific composite outcomes in patients with CKD and diabetes forest plot. Figure S9: Albuminuria reduction pairwise meta‐analysis forest plot. Figure S10: Albuminuria reduction with SGLT2i pairwise meta‐analysis forest plot. Figure S11: Albuminuria reduction with nsMRA pairwise meta‐analysis forest plot. Figure S12: Trial‐level association between albuminuria reduction and kidney outcomes. Figure S13: Trial‐level association between albuminuria reduction and kidney outcomes stratified by class. Table S1: Composite outcome component across trials. Table S2: Comparison of key effect modifiers across trials to assess the plausibility of the transitivity assumption. Table S3: Network connectivity and design structure. Table S4: Heterogeneity statistics and design‐specific Q decomposition. Table S5: CINeMA confidence assessment. Table S6: Comparison with prior network meta‐analysis. [file EDM2-9-e70285-s001.docx]

**Supplementary Appendix A1**

**Title:** Placebo-Referenced Class-Level Treatment Effects on Chronic Kidney Disease Progression in Patients with Diabetes: A Network Meta-analysis

**Short title:** Placebo-Referenced Effects on CKD Progression

**Author details:**

Ravi Kumar Pandey*^1^, MBBS; Maryam Imran^2^, MBBS; Ishba Manal^3^, MBBS; Muhammad Ahmad^4^, MBBS; Aliya Noor^5^, MBBS; Ali Rohan^6^, MBBS

Affiliations:

^1^Nepalgunj Medical College, Nepalgunj, Nepal (Internal Medicine)

^2^Fatima Jinnah Medical University, Lahore, Pakistan (Internal Medicine)

_3_Dow University of Health Sciences, Karachi, Pakistan (Internal Medicine)

^4^Sharif Medical City, Lahore, Pakistan (Internal Medicine)

^5^King Edward Medical University, Lahore, Pakistan (Internal Medicine)

^6^ The University of Lahore, University College of Medicine and Dentistry, Lahore, Pakistan (Internal Medicine)

Emails:

[^1^pandeyravi0211@gmail.com](mailto:1pandeyravi0211@gmail.com)

^2^ [maryamimran64@gmail.com](mailto:maryamimran64@gmail.com)

^3^ [faiqishba@gmail.com](mailto:faiqishba@gmail.com)

^4^  [drahmad.md24@gmail.com](mailto:drahmad.md24@gmail.com)

^5^  [aliyanoor7866@gmail.com](mailto:aliyanoor7866@gmail.com)

^6^ [ali.rohan789@gmail.com](mailto:ali.rohan789@gmail.com)

ORCID ID

^1^0009-0000-7355-9532

^2^0009-0001-0166-1412

^3^0000-0003-1299-9045

^4^0009-0009-9603-6166

^5^0009-0000-5376-2437

^6^0009-0000-9986-3495

**Corresponding Author Detail**

* Ravi Kumar Pandey

Affiliation: Nepalginj Medical College, Nepalgunj, Nepal

Email: [pandeyravi0211@gmail.com](mailto:pandeyravi0211@gmail.com)

Mail address: Gaur, Nepal, 44500

This supplement contains additional methods, tables, and figures supporting the manuscript.

| **Search string** |
| --- |
| **Section 1. PRISMA Flow Diagram, Composite Outcome Components, Publication Bias, Network Geometry, Model Diagnostics, and Confidence Assessments** |
| **Section 2. Full Efficacy Results** |
| **Section 3. Albuminuria and Surrogate Analyses Results** |

**Abbreviations:** CKD = chronic kidney disease; eGFR = estimated glomerular filtration rate; ET antagonist = endothelin receptor antagonist; GLP-1RA = glucagon-like peptide-1 receptor agonist; nsMRA = nonsteroidal mineralocorticoid receptor antagonist; RAS inhibitor = renin-angiotensin system inhibitor; SGLT2i = sodium-glucose cotransporter-2 inhibitor; UACR = urinary albumin-to-creatinine ratio.

**Search String**

- PUBMED

("Chronic Kidney Disease"[MeSH Terms] OR "Diabetic Nephropathies"[MeSH Terms] OR "chronic kidney disease"[tiab] OR CKD[tiab] OR "diabetic kidney disease"[tiab] OR "diabetic nephropathy"[tiab] OR "chronic renal insufficiency"[tiab]) AND ("Diabetes Mellitus"[MeSH Terms] OR diabetes[tiab] OR diabetic[tiab])AND ("Sodium-Glucose Transporter 2 Inhibitors"[MeSH Terms] OR "Glucagon-Like Peptide 1 Receptor Agonists"[MeSH Terms] OR "Angiotensin-Converting Enzyme Inhibitors"[MeSH Terms] OR "Angiotensin Receptor Antagonists"[MeSH Terms] OR "Mineralocorticoid Receptor Antagonists"[MeSH Terms] OR "SGLT2 inhibitor*"[tiab] OR canagliflozin[tiab] OR dapagliflozin[tiab] OR empagliflozin[tiab] OR "GLP-1 receptor agonist*"[tiab] OR liraglutide[tiab] OR semaglutide[tiab] OR dulaglutide[tiab] OR "ACE inhibitor*"[tiab] OR "angiotensin receptor blocker*"[tiab] OR losartan[tiab] OR irbesartan[tiab] OR valsartan[tiab] OR telmisartan[tiab] OR candesartan[tiab] OR finerenone[tiab] OR spironolactone[tiab] OR eplerenone[tiab]) AND ("Disease progression"[MeSH Terms] OR progress[tiab] OR "renal outcome*"[tiab] OR "kidney outcome*"[tiab] OR "eGFR decline*"[tiab] OR "renal function decline"[tiab]) AND (randomized controlled trial[pt] OR controlled clinical trial[pt] OR randomized[tiab]) NOT (animals[mh] NOT humans[mh])

- Embase

('chronic kidney disease'/exp OR 'chronic kidney disease' OR CKD OR 'diabetic nephropathy'/exp OR 'diabetic nephropathy' OR 'chronic renal insufficiency'/exp OR 'chronic renal insufficiency' AND ('diabetes mellitus'/exp OR 'diabetes mellitus' OR 'dm type 2' AND ('sodium glucose cotransporter 2 inhibitor'/exp OR 'sodium glucose cotransporter 2 inhibitor' OR 'glucagon like peptide 1 receptor agonist'/exp OR 'glucagon like peptide 1 receptor agonist' OR 'angiotensin receptor antagonist'/exp OR 'angiotensin receptor antagonist' OR 'mineralocorticoid receptor antagonist'/exp OR 'mineralocorticoid receptor antagonist' OR 'empagliflozin'/exp OR empagliflozin OR 'dapagliflozin'/exp OR dapagliflozin OR 'canagliflozin'/exp OR canagliflozin OR 'liraglutide'/exp OR liraglutide OR 'semaglutide'/exp OR semaglutide OR 'dulaglutide'/exp OR dulaglutide OR 'tirzepatide'/exp OR tirzepatide OR 'losartan'/exp OR losartan OR 'valsartan'/exp OR valsartan OR 'telmisartan'/exp OR telmisartan OR 'spironolactone'/exp OR spironolactone OR 'eplerenone'/exp OR eplerenone OR 'finerenone'/exp OR finerenone) AND ('disease progression'/exp OR 'disease progression' OR 'renal outcome'/exp OR 'renal function decline' OR 'kidney outcome'/exp OR 'kidney outcome' OR 'eGFR decline' OR 'renal function decline') AND 'randomized controlled trial'/de

- Cochrane CENTRAL

("chronic kidney disease" OR CKD OR "chronic renal disease" OR "chronic renal insufficiency" OR "kidney failure chronic" OR "diabetic nephropathy" OR "diabetic kidney disease" OR "diabetic renal disease") AND ("sodium glucose cotransporter 2 inhibitor*" OR dapagliflozin OR empagliflozin OR canagliflozin OR "GLP-1 receptor agonist" OR "glucagon like peptide 1 receptor agonist*" OR liraglutide OR semaglutide OR dulaglutide OR "ACE inhibitor*" OR enalapril OR lisinopril OR ramipril OR captopril OR "angiotensin receptor blocker*" OR losartan OR valsartan OR irbesartan OR candesartan OR telmisartan OR olmesartan OR "mineralocorticoid receptor antagonist*" OR finerenone OR spironolactone OR eplerenone) AND “renal outcome” OR “disease progression” OR "kidney outcome" OR "eGFR decline")

**Section 1. PRISMA Flow Diagram, Composite Outcome Components, Publication Bias, Network Geometry, Model Diagnostics, and Confidence Assessments**

*
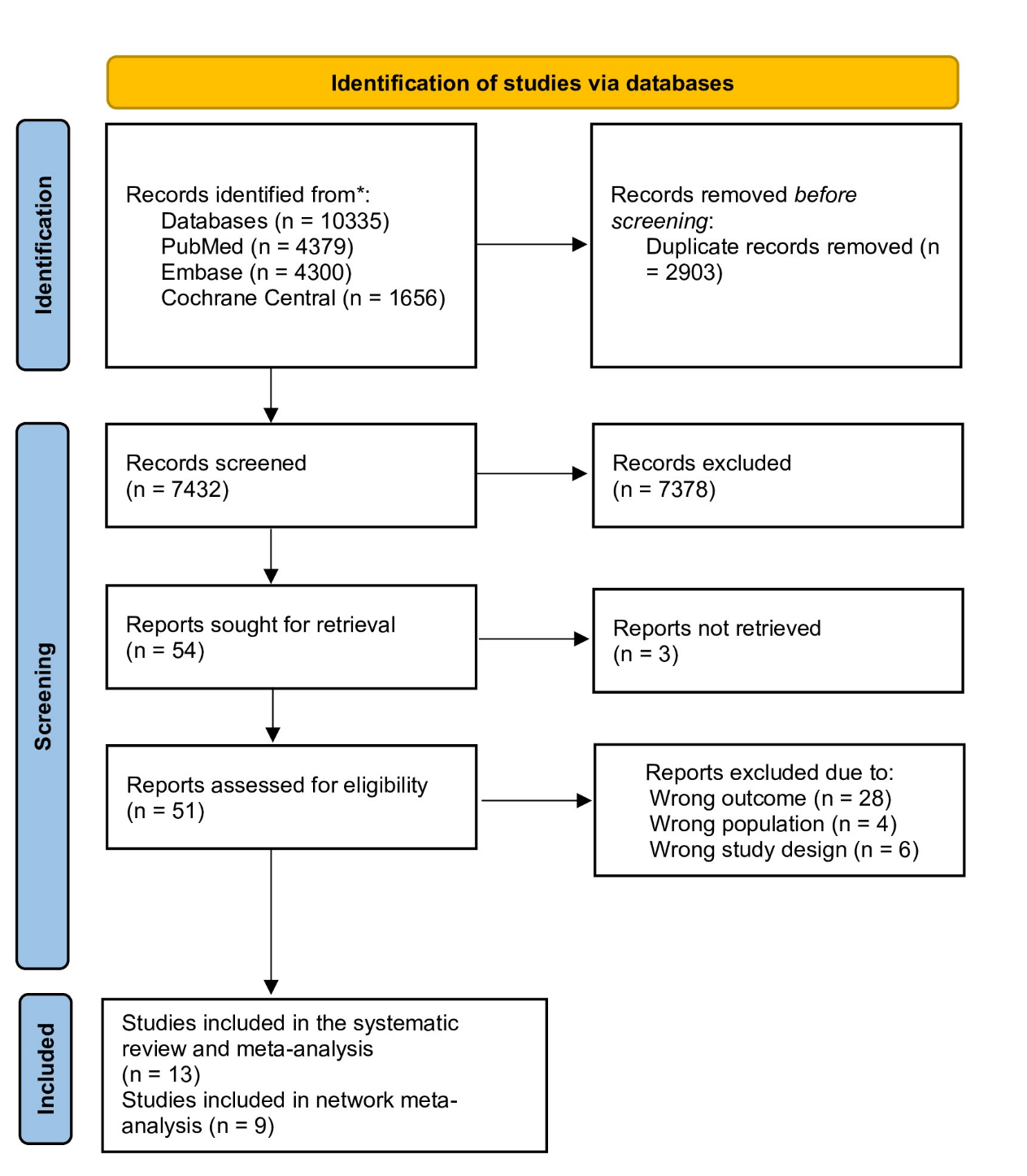
*

**Figure S1. PRISMA Flow Diagram**

*Table S1. Composite Outcome Component Across Trials*

| Trials | Diabetic population only | CV death as a component of the composite | Kidney function decline threshold | Renal failure/ESKD included |
| --- | --- | --- | --- | --- |
| CREDENCE | Yes | Yes | ≥50 | Yes |
| DAPA-CKD | Mixed | Yes | ≥50 | Yes |
| EMPA-KIDNEY | Mixed | Yes | ≥40% | Yes |
| FIDELIO-DKD | Yes | No | ≥40% | Yes |
| FIGARO-DKD | Yes | No | ≥40% | Yes |
| FLOW | Yes | Yes | ≥50 | Yes |
| SONAR | Yes | No | ≥50 | Yes |
| IDNT | Yes | Yes | Doubling serum creatinine (~57% eGFR decline) | Yes |
| RENAAL | Yes | Yes | Doubling serum creatinine (~57% eGFR decline) | Yes |

***** This table summarizes key differences in the definitions of the primary kidney composite outcome across included trials. Although the individual components differed, all composite outcomes captured the common biological construct of progressive CKD by incorporating sustained kidney function decline together with clinically meaningful kidney failure outcomes. These differences were considered during the interpretation of pooled treatment effects and explored through sensitivity analyses using renal-specific composite outcomes.

† CV = cardiovascular; eGFR = estimated glomerular filtration rate; ESKD = end-stage kidney disease; UACR = urinary-albumin-to-creatinine ratio.

*
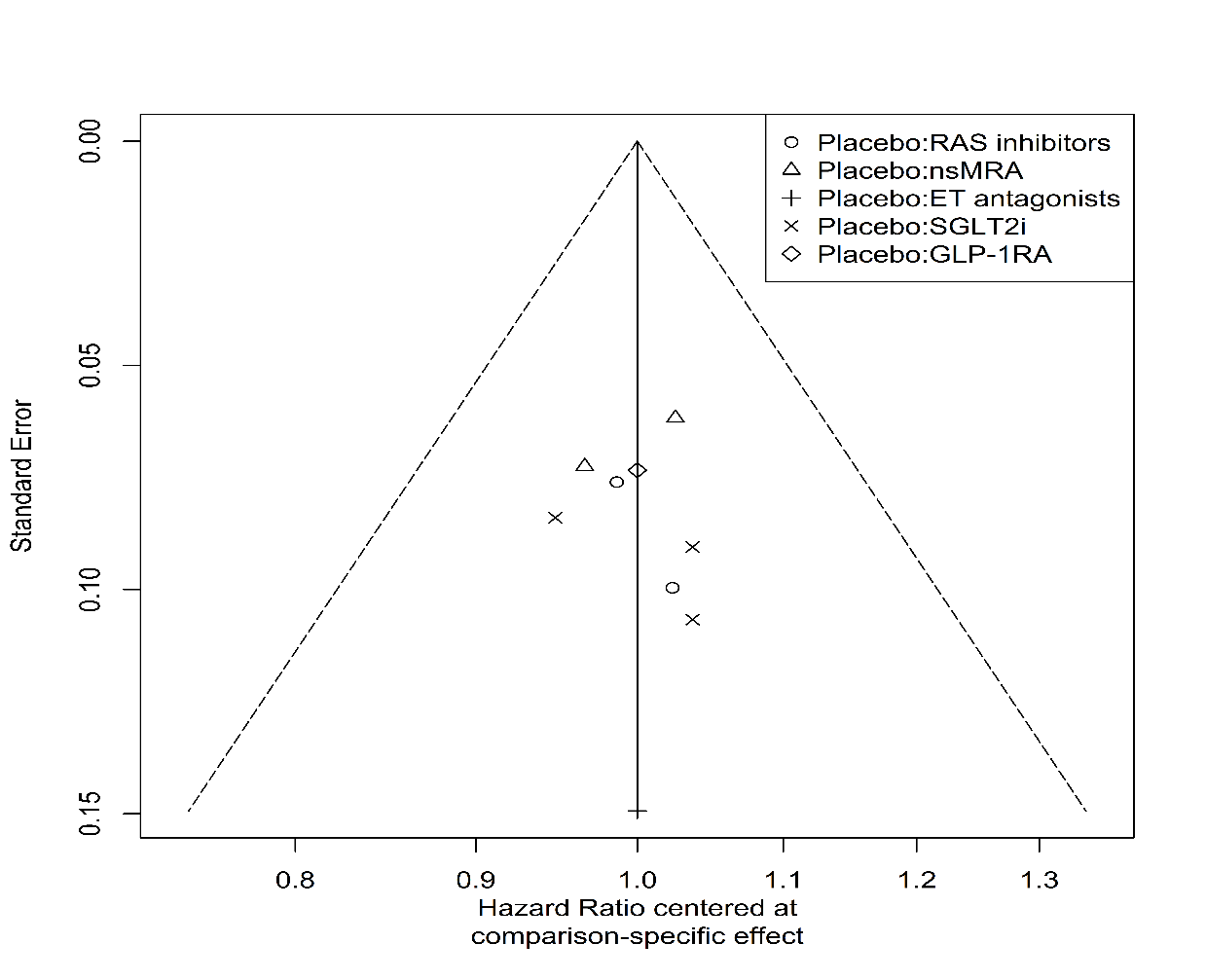
* **Figure S2. Comparison Adjusted Funnel Plot**

Funnel plot of treatment effects from the network meta-analysis. Each point represents an individual study comparison, with the horizontal axis showing comparison-adjusted hazard ratios (HRs) and the vertical axis showing standard error. The solid vertical line represents the pooled network effect, and dashed lines indicate pseudo 95% confidence limits. Visual inspection did not suggest substantial small-study effects or publication bias, although interpretation is limited by the small number of included studies.

ET antagonist = endothelin receptor antagonist; nsMRA = nonsteroidal mineralocorticoid receptor antagonist; GLP-1RA = glucagon-like peptide-1 receptor agonist; RAS inhibitor = renin-angiotensin system inhibitor; SGLT2i = sodium-glucose cotransporter-2 inhibitor.

*
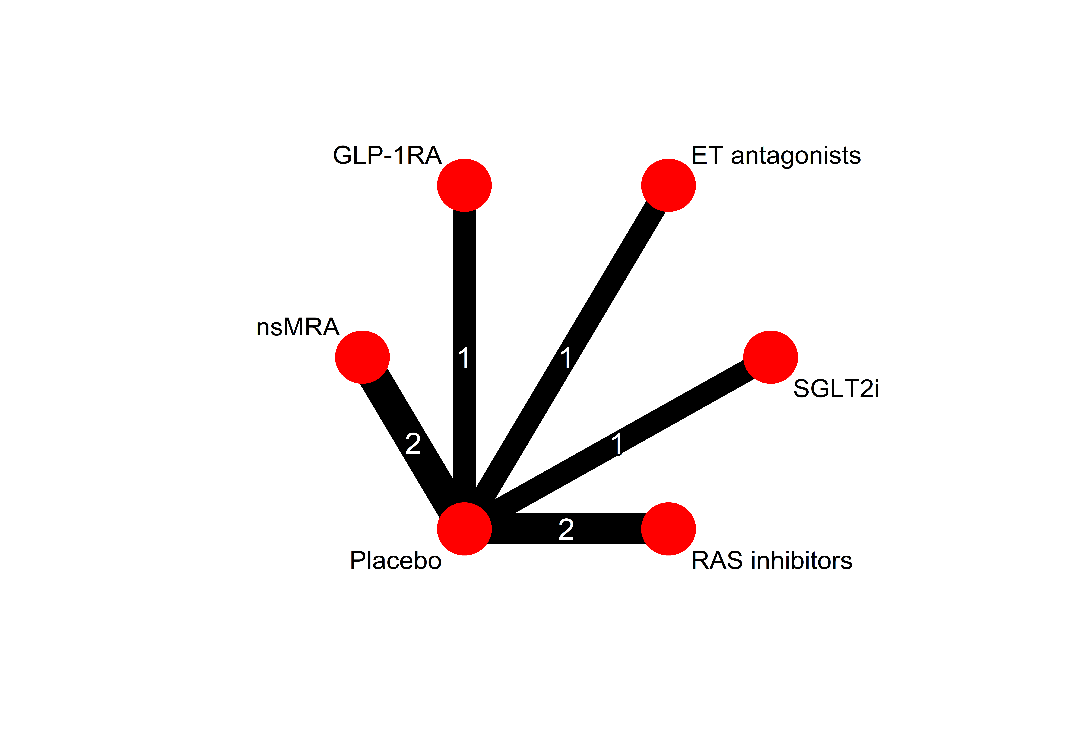
*

**Figure S3. Network Plot for Primary Composite Outcome in Trials Enrolling Patients with CKD and Diabetes**

Network geometry restricted to trials enrolling patients with diabetes and chronic kidney disease. Nodes represent treatment classes evaluated across trials, and edges indicate direct head-to-head comparisons between classes and placebo. Edge thickness represents the number of trials used to compare treatment classes.

*Table S2. Comparison of Key Effect Modifiers Across Trials to Assess the Plausibility of the Transitivity Assumption*

| Trial | Drug class | Year of publication | Kidney function eligibility | Albuminuria/proteinuria eligibility | Baseline kidney function | Baseline albuminuria (median UACR or proteinuria) | Background RAS inhibitor use (%) | Background SGLT2i use (%) | Established cardiovascular disease (%) | Follow up (years) | Special design features |
| --- | --- | --- | --- | --- | --- | --- | --- | --- | --- | --- | --- |
| CREDENCE | SGLT2i | 2019 | eGFR 30 to <90 mL/min/1.73 m² | UACR300 to 5000 | 56.2 | 927 | 99.9 | No | 50.4 | 2.6 | None |
| DAPA-CKD | SGLT2i | 2020 | eGFR 25 to <75 mL/min/1.73 m² | UACR 200 to 5000 | 43.1 | 950 | 98.5 | No | 37.4 | 2.4 | Mixed CKD population; diabetic subgroup analyzed |
| EMPA-KIDNEY | SGLT2i | 2022 | eGFR 25 to <90 mL/min/1.73 m² | UACR >200 | 37.3 | 329 | 84.6 | No | 26.7 | 2.0 | Mixed CKD population; diabetic subgroup analyzed |
| FIDELIO-DKD | nsMRA | 2020 | eGFR 25 to <75 mL/min/1.73 m² | UACR 300 to 5000 | 44.3 | 852 | 99.6 | 4.6 | 45.9 | 2.6 | None |
| FIGARO-DKD | nsMRA | 2021 | eGFR 25 to <90 mL/min/1.73 m² | UACR 300 to 5000 | 67.8 | 308 | 99.9 | 8.4 | 45.3 | 3.4 | None |
| FLOW | GLP-1RA | 2024 | eGFR 25 to <75 mL/min/1.73 m² | UACR 100 to 5000 | 47 | 567.6 | 95.3 | 15.6 | 42.1 | 3.4 | None |
| SONAR | ET antagonist | 2019 | eGFR 25 to <75 mL/min/1.73 m² | UACR 300 to 5000 | 43.8 | 801 | 100 | 1.45 | Not reported | 3.7 | Enrichment design |
| RENAAL | RAS inhibitor | 2001 | Serum creatinine between 1.3 and 3 mg/dL | UACR >300 | Serum creatinine 1.9 mg/dL | 1249 | Not receiving RAS inhibition at randomization | Not available (predated SGLT2i) | 21 | 3.4 | Historical treatment-initiation trial |
| IDNT | RAS inhibitor | 2001 | Serum creatinine between 1 and 3 mg/dL | >900 mg proteinuria in 24 hour | Serum creatinine 1.6 mg/dL | 1900mg in 24 hours | Not receiving RAS inhibition at randomization | Not available (predated SGLT2i) | 28 | 2.6 | Historical treatment-initiation trial |

* Prespecified study- and population-level effect modifiers relevant to the transitivity assumption are summarized for each included trial. These variables were considered when assessing the plausibility and interpretability of indirect comparisons.

† CKD = chronic kidney disease; ET antagonist = endothelin receptor antagonist; eGFR = estimated glomerular filtration rate; GLP-1RA = glucagon-like peptide-1 receptor agonist; nsMRA = nonsteroidal mineralocorticoid receptor antagonist; RAS inhibitor = renin-angiotensin system inhibitor; SGLT2i = sodium-glucose cotransporter-2 inhibitor; UACR = urinary albumin to creatinine ratio.

*Table S3. Network Connectivity and Design Structure*

| Analysis | Number of studies (k) | Number of pairwise comparisons (m) | Number of treatments (n) | Number of designs (d) | Number of connections |
| --- | --- | --- | --- | --- | --- |
| Primary composite outcome | 9 | 9 | 6 | 5 | 1 |
| Trials Enrolling Patients with CKD and Diabetes | 7 | 7 | 6 | 5 | 1 |
| Contemporary trials only analysis | 7 | 7 | 5 | 4 | 1 |
| eGFR ≥50% decline outcome analysis | 6 | 6 | 5 | 4 | 1 |
| Exploratory renal composite outcome analysis | 6 | 6 | 6 | 5 | 1 |

* All networks formed a single connected component with a star-shaped structure centered on the placebo. CKD = chronic kidney disease; NMA = network meta-analysis.

*Table S4. Heterogeneity Statistics and Design-Specific Q Decomposition*

| Analysis | I squared | Tau- squared | Q Total | Df | P-value | Q between |
| --- | --- | --- | --- | --- | --- | --- |
| Primary composite outcome | 0 | 0 | 1.15 | 4 | 0.8863 | 0 |
| Trials Enrolling Patients with CKD and Diabetes | 0 | 0 | 0.47 | 2 | 0.7905 | 0 |
| Contemporary therapy classes only analysis | 0 | 0 | 1.07 | 3 | 0.7854 | 0 |
| ≥50% eGFR reduction analysis | 0 | 0 | 0.65 | 2 | 0.7215 | 0 |
| Exploratory renal composite outcome | 0 | 0 | 0.39 | 1 | 0.5345 | 0 |

* Q between represents the design-by-treatment interaction component. Because networks were star-shaped without closed loops, Q between was structurally zero. eGFR = estimated glomerular filtration rate.

*Table S5. CINeMA Confidence Assessment*

| Comparison | Within-study bias | Reporting bias | Indirectness | Imprecision | Heterogeneity | Incoherence | Confidence |
| --- | --- | --- | --- | --- | --- | --- | --- |
| ET antagonist vs Placebo | Some concerns | Some concerns | Major concerns | No concerns | No concerns | Not assessable | Low |
| GLP-1RA vs Placebo | Some concerns | Some concerns | No concerns | No concerns | No concerns | Not assessable | Moderate |
| Placebo vs RAS inhibitors | No concerns | Some concerns | Major concerns | No concerns | No concerns | Not assessable | Low |
| Placebo vs SGLT2i | No concerns | Some concerns | No concerns | No concerns | No concerns | Not assessable | High |
| Placebo vs nsMRA | No concerns | Some concerns | Some concerns | No concerns | No concerns | Not assessable | Moderate |
| ET antagonist vs GLP-1RA | Some concerns | Some concerns | No concerns | Major concerns | No concerns | Not assessable | Low |
| ET antagonist vs RAS inhibitors | No concerns | Some concerns | Major concerns | Major concerns | No concerns | Not assessable | Very low |
| ET antagonist vs SGLT2i | No concerns | Some concerns | Major concerns | Major concerns | No concerns | Not assessable | Very low |
| ET antagonist vs nsMRA | No concerns | Some concerns | Some concerns | Major concerns | No concerns | Not assessable | Low |
| GLP-1RA vs RAS inhibitors | No concerns | Some concerns | No concerns | Major concerns | No concerns | Not assessable | Low |
| GLP-1RA vs SGLT2i | No concerns | Some concerns | No concerns | Major concerns | No concerns | Not assessable | Low |
| GLP-1RA vs nsMRA | No concerns | Some concerns | No concerns | Major concerns | No concerns | Not assessable | Low |
| RAS inhibitors vs SGLT2i | No concerns | Some concerns | Major concerns | No concerns | Major concerns | Not assessable | Very low |
| RAS inhibitors vs. NSMRA | No concerns | Some concerns | Some concerns | Major concerns | No concerns | Not assessable | Low |
| SGLT2i vs nsMRA | No concerns | Some concerns | Some concerns | No concerns | No concerns | Not assessable | Moderate |

* Confidence in network estimates was assessed using the CINeMA (Confidence in Network Meta-Analysis) framework across six domains: within-study bias, reporting bias, indirectness, imprecision, heterogeneity, and incoherence. Judgments for each domain were categorized as no concerns, some concerns, or major concerns, and were combined to derive an overall confidence rating (high, moderate, low, or very low) for each comparison.

**Section S2: Full Efficacy Results**

*
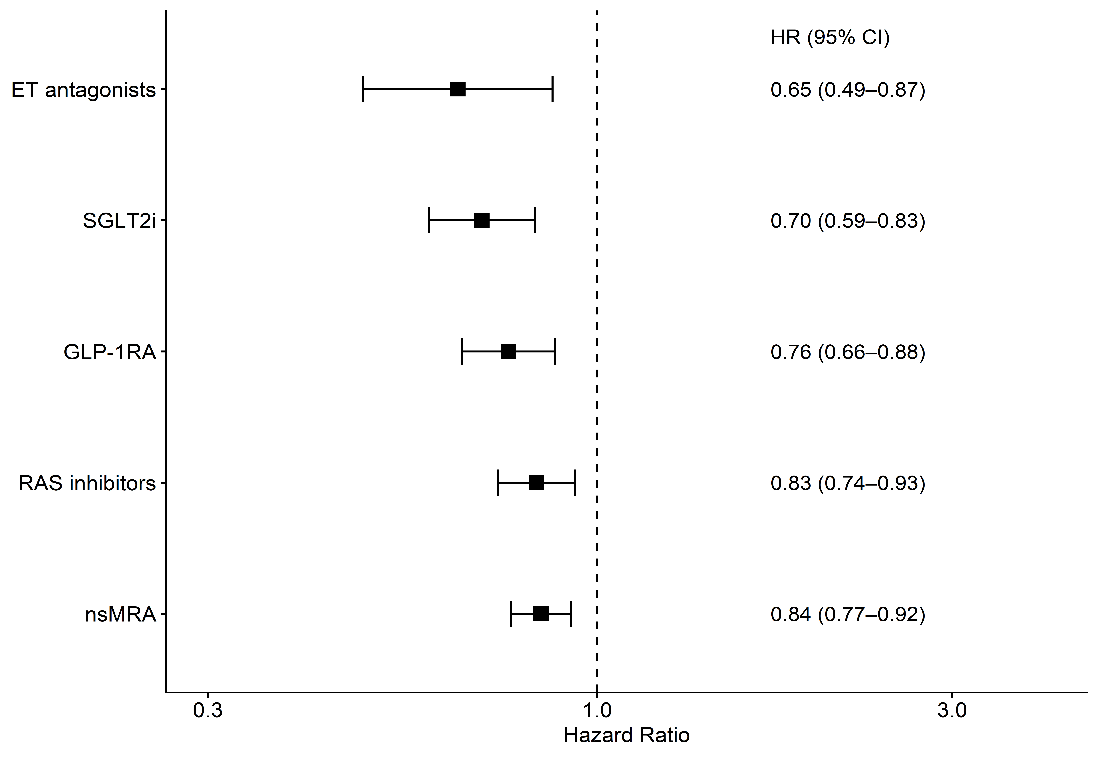
*

**Figure S4. Trials Enrolling Patients with CKD and Diabetes Forest Plot: Random Effects Model**

Forest plot of treatment effects from network meta-analysis restricted to trials exclusively enrolling patients with diabetes. Hazard ratios (HRs) with 95% confidence intervals (CIs) are presented. This analysis assesses the consistency of treatment effects within a homogeneous diabetic population.

ET antagonist = endothelin receptor antagonist; GLP-1RA = glucagon-like peptide-1 receptor agonist; nsMRA = nonsteroidal mineralocorticoid receptor antagonist; RAS inhibitor = renin-angiotensin system inhibitor; SGLT2i = sodium-glucose cotransporter-2 inhibitor.

*
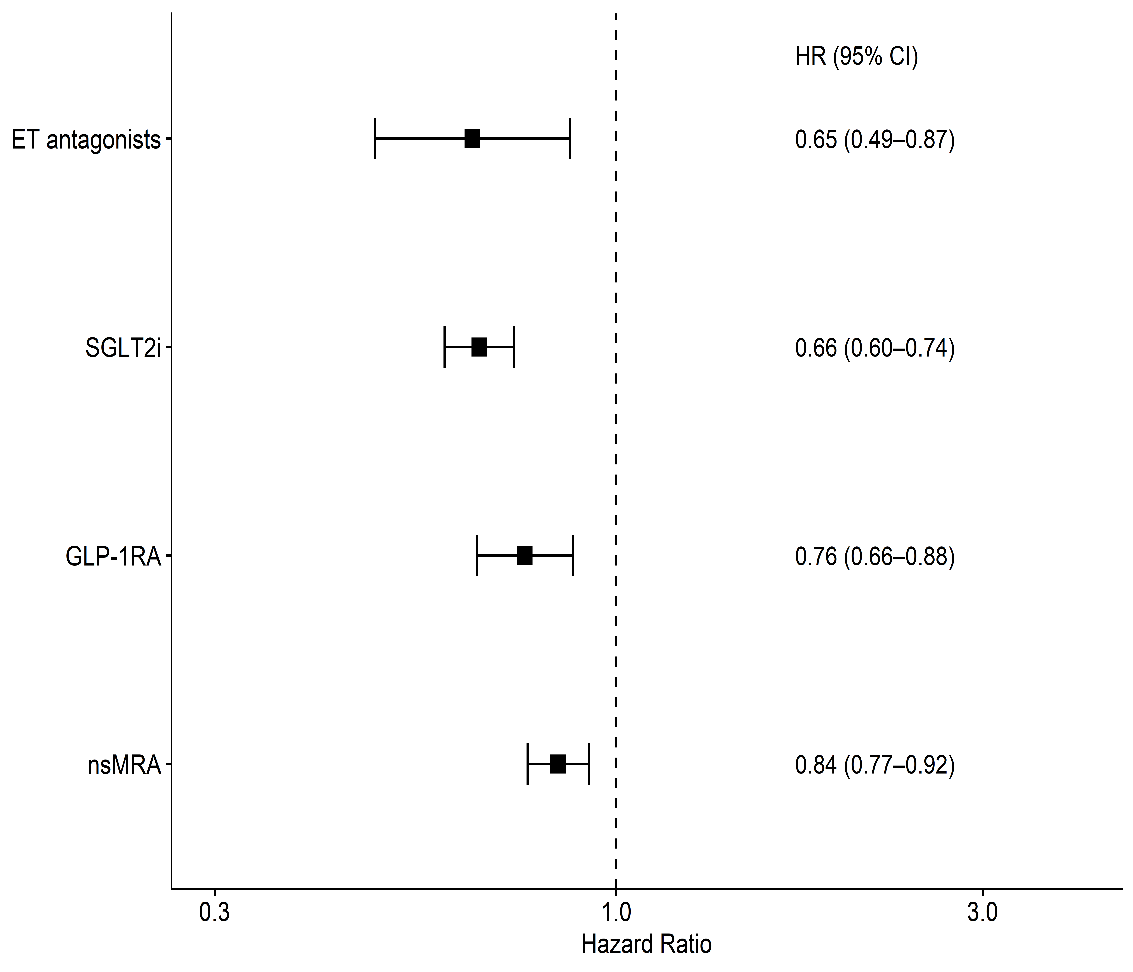
*

**Figure S5. Contemporary Trials Only Forest Plot: Random Effects Model**

Forest plot of class-level treatment effects restricted to contemporary trials evaluating newer therapeutic classes, excluding legacy renin-angiotensin system (RAS) inhibitor trials. Hazard ratios (HRs) with 95% confidence intervals (CIs) are presented.

*
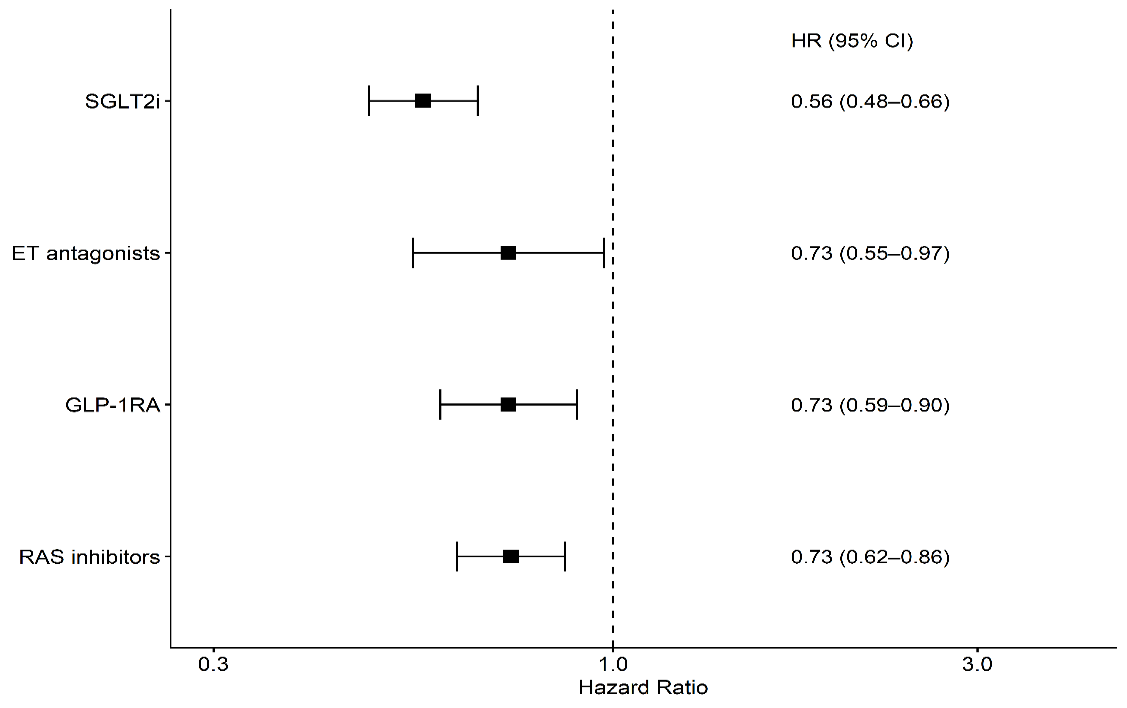
*

**Figure S6. ≥50% Decline in eGFR Outcome Forest Plot: Random Effects Model**

Forest plot of class-level treatment effects on composite outcome with trials containing ≥50% decline in eGFR as a composite outcome, derived from network meta-analysis. Hazard ratios (HRs) with 95% confidence intervals (CIs) are shown. This analysis represents an alternative, more stringent definition of kidney disease progression.

*
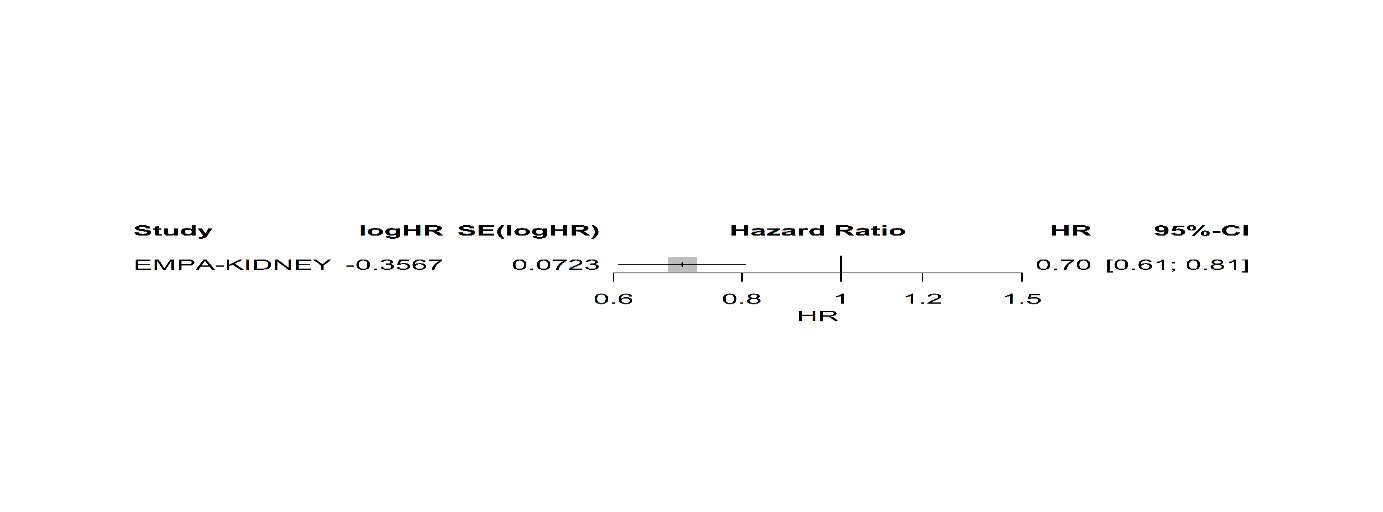
*

A

*
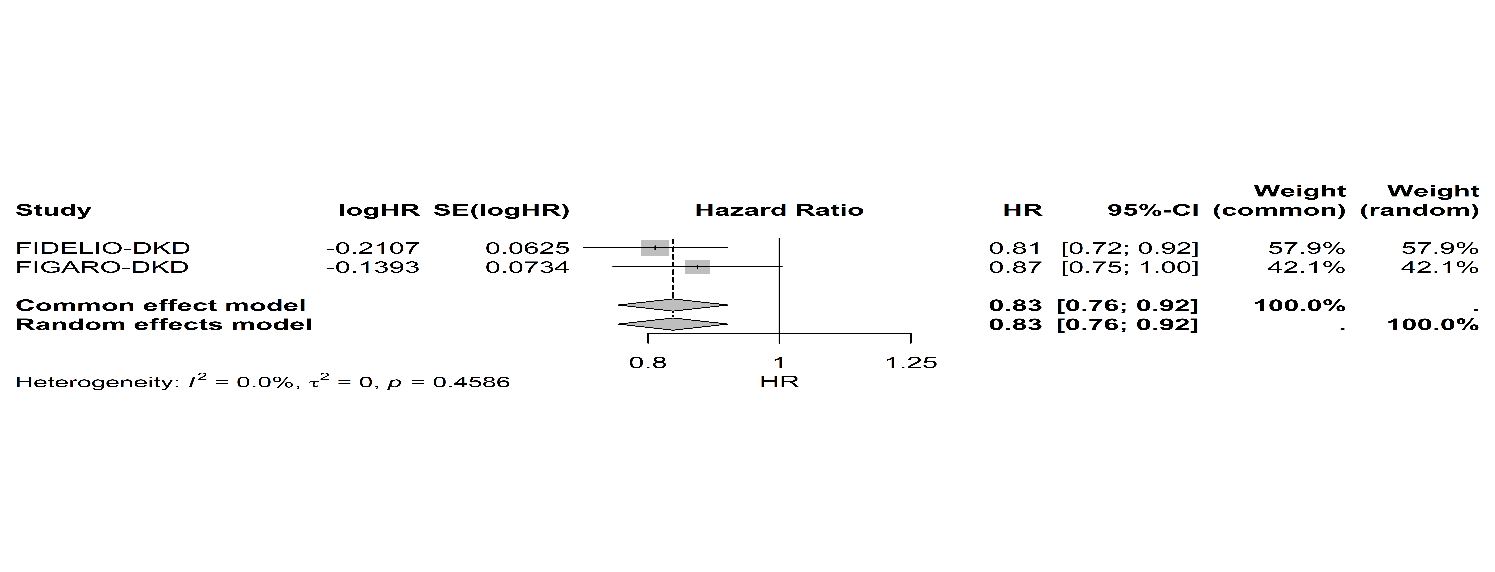
*

B

**Figure S7. ≥40% Decline in eGFR Outcome Pairwise Meta-analysis Forest Plot**

Forest plot of the treatment effects of A. sodium glucose co-transporter 2 inhibitors (SGLT2i) and B. non-steroidal mineralocorticoid receptor antagonists (nsMRA) on ≥40% decline in eGFR derived from conventional pairwise random-effects meta-analysis. Hazard ratios (HRs) with 95% confidence intervals (CIs) are shown. Network meta-analysis was not feasible due to insufficient connectivity.


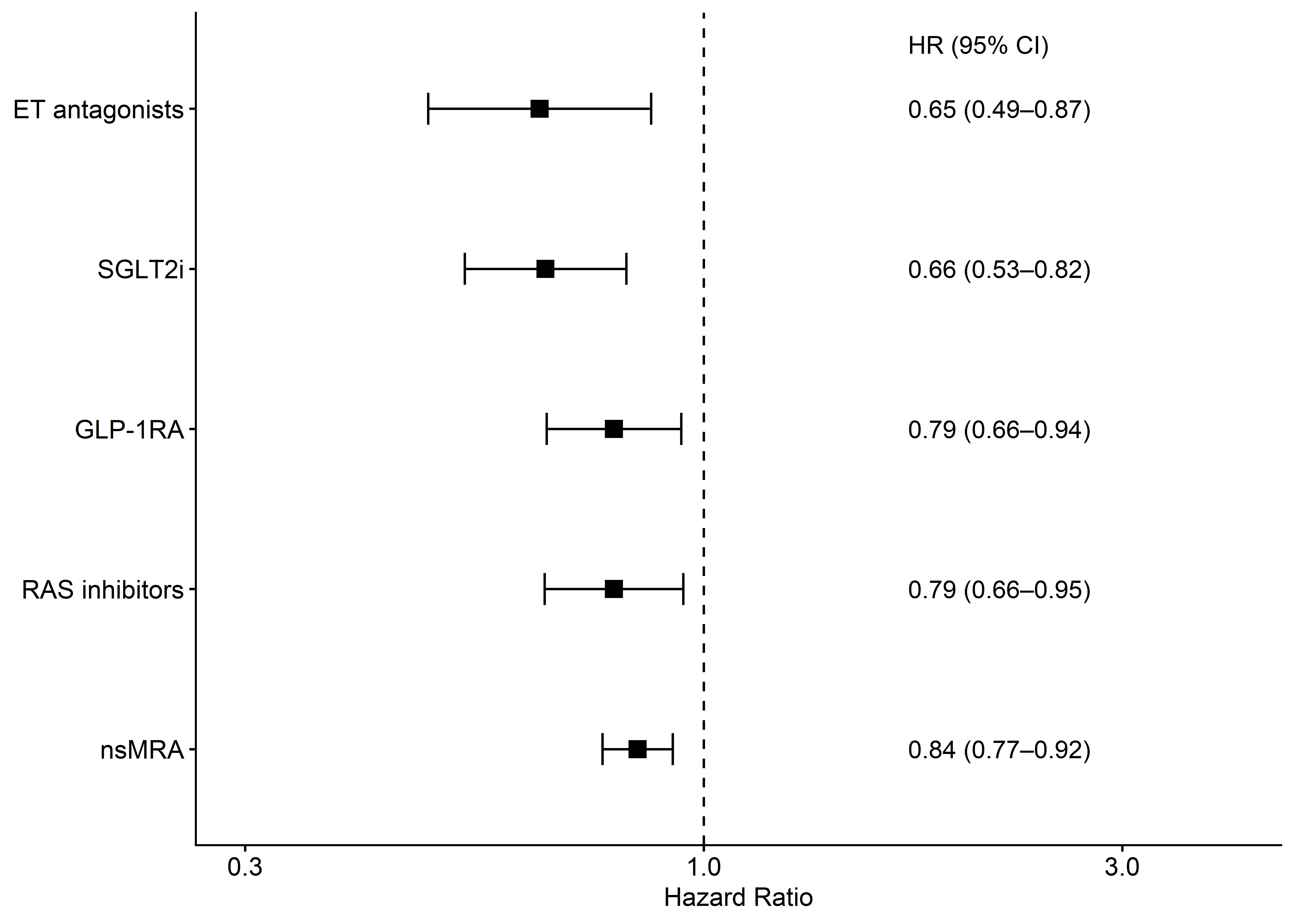


**Figure S8. Exploratory Renal Specific Composite Outcomes in Patients with CKD and Diabetes Forest Plot**

A forest plot of renal-specific composite outcome in patients with chronic kidney disease (CKD) and diabetes is shown. Effect estimates are presented as hazard ratios (HRs) with 95% confidence intervals (CIs). Renal-specific composite estimates were derived from primary or secondary trial endpoints according to individual study reporting. Hazard ratio <1 favors active treatment.

ET antagonist = endothelin receptor antagonist; GLP-1RA = glucagon-like peptide-1 receptor agonist; nsMRA = nonsteroidal mineralocorticoid receptor antagonist; RAS inhibitor = renin-angiotensin system inhibitor; SGLT2i = sodium-glucose cotransporter-2 inhibitor.

**Section 3. Albuminuria and Surrogate Analyses Results**

*Section 3.1* *Albuminuria Analysis*

Across six randomized trials, treatments were associated with a reduction in urinary albumin-to-creatinine ratio (UACR). In random-effects meta-analysis, the pooled ratio of geometric means was 0.68 (95% CI 0.66–0.69). Statistical heterogeneity was low (I² = 0.1%; Q = 4.45, p = 0.480), although the limited number of studies may reduce the power to detect between-study variability.

**Class-stratified analyses**

SGLT2i was associated with a pooled ratio of 0.67 (95% CI 0.63–0.71), while nsMRA demonstrated a pooled ratio of 0.68 (95% CI 0.67–0.70). A single GLP-1RA trial showed a comparable effect size (0.68, 95% CI 0.62–0.75).

Meta-regression by drug class did not demonstrate a significant difference in treatment effect (p for interaction = 0.730). Within-class heterogeneity was low among nsMRA trials (I²=0%), while the moderate heterogeneity observed in the SGLT2i subgroup (I²=42%) likely reflects instability related to the small number of studies.

These analyses were based on a limited number of studies and should be interpreted as exploratory.

*
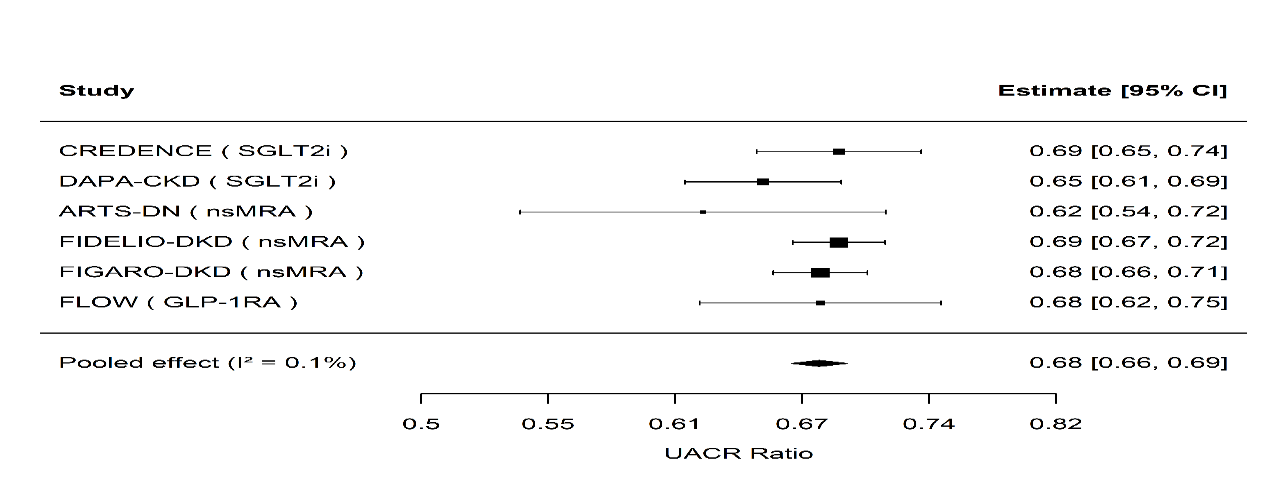
*

**Figure S9. Albuminuria Reduction Pairwise Meta-analysis Forest Plot**

The forest plot represents treatment effects on urinary albumin-to-creatinine ratio (UACR) expressed as ratios of geometric means. Values <1 indicate a reduction in proteinuria. Squares represent individual study estimates weighted by inverse variance, with horizontal lines indicating 95% confidence intervals (CIs). The diamond represents the pooled estimate from a random-effects model. Treatment was associated with a consistent reduction in UACR across trials (pooled ratio 0.68, 95% CI 0.66–0.69), with negligible heterogeneity (I² = 0.1%).

*
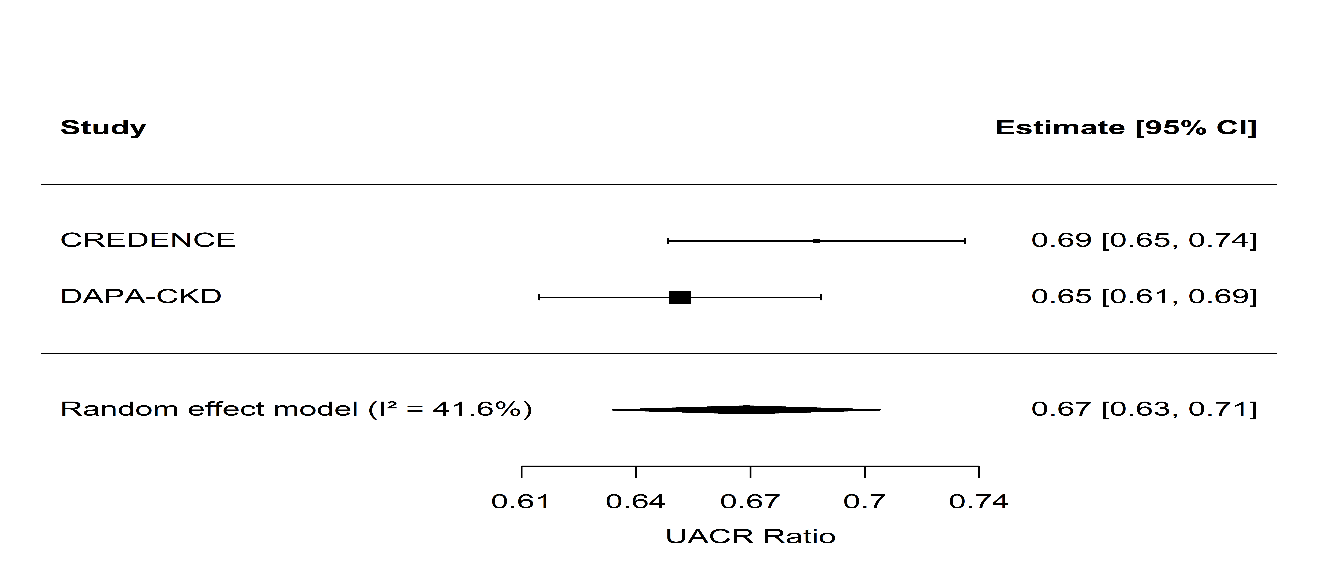
*

**Figure S10. Albuminuria Reduction with SGLT2i Pairwise Meta-analysis Forest Plot**

Forest plot of treatment effects on urinary albumin-to-creatinine ratio (UACR) among sodium glucose co-transporter 2 inhibitor (SGLT2i) trials, expressed as ratios of geometric means. Values <1 indicate a reduction in albuminuria with active treatment. Heterogeneity was moderate, though interpretation is limited by the small number of included studies.

*
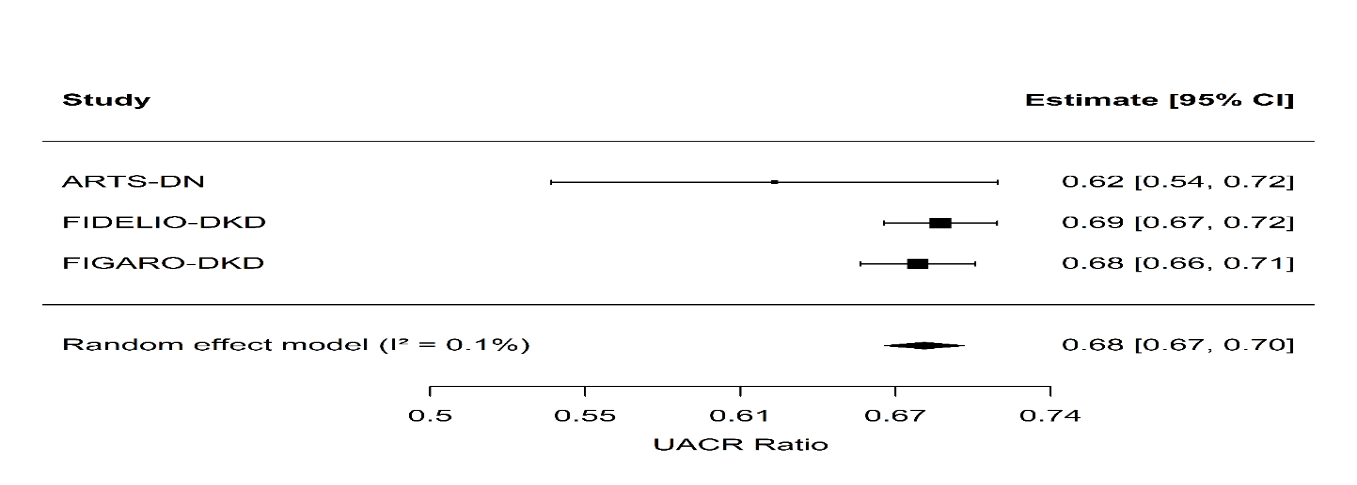
*

**Figure S11. Albuminuria Reduction with nsMRA Pairwise Meta-analysis Forest Plot**

Forest plot of treatment effects on urinary albumin-to-creatinine ratio (UACR) among nonsteroidal mineralocorticoid receptor antagonist (nsMRA) trials, expressed as ratios of geometric means. Values <1 indicate a reduction in albuminuria with active treatment.

*Section 3.2 Surrogate Endpoint Analysis*

Across included trials, weighted meta-regression demonstrated no statistically significant association between treatment-induced changes in albuminuria and treatment effects on kidney outcomes. The estimated slope was small and non-significant (β = 0.34, p = 0.8500), with minimal explained variance (R² = 0.007).

Visual inspection of the regression plot showed a near-flat relationship, with substantial dispersion of trial-level estimates and wide confidence intervals, indicating high uncertainty. However, given the limited number of trials and the use of aggregated trial-level data, these findings should be interpreted with caution.

**Stratification by pharmacologic class**

Among sodium glucose co-transporter 2 inhibitor (SGLT2i) trials, a directional association between albuminuria reduction and kidney outcomes was noted, whereas other classes demonstrated less consistent patterns. Given the limited number of trials per class, these findings are exploratory and hypothesis-generating, and no formal inference was performed.

*
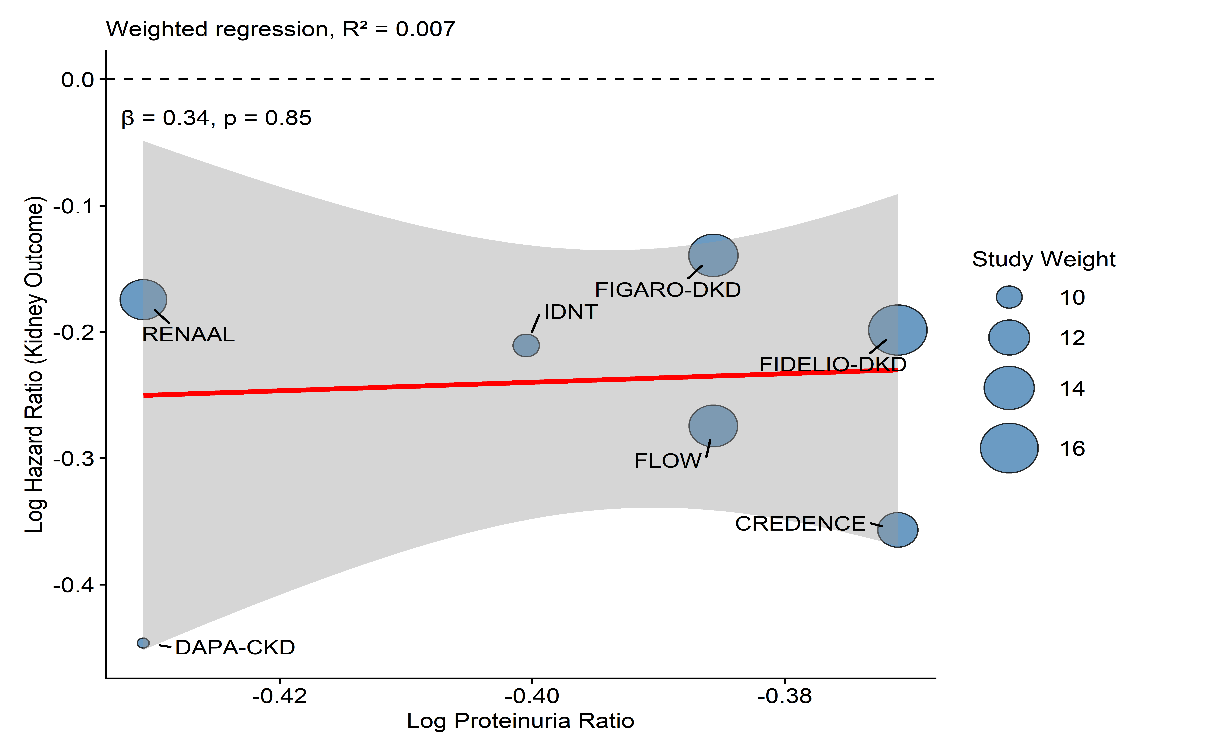
*

**Figure S12. Trial-Level Association between Albuminuria Reduction and Kidney Outcomes**

Bubble plot showing the relationship between log-transformed proteinuria or albuminuria ratio and log hazard ratio (HR) for kidney outcomes across included trials. Bubble size is proportional to study weight. The red line represents the weighted linear regression, with the shaded region indicating the 95% confidence interval (CI). The horizontal dashed line represents a null effect (log HR = 0). Weighted meta-regression demonstrated no significant association (β = 0.34, p = 0.85; R² = 0.007), indicating limited explanatory value within this dataset.

*
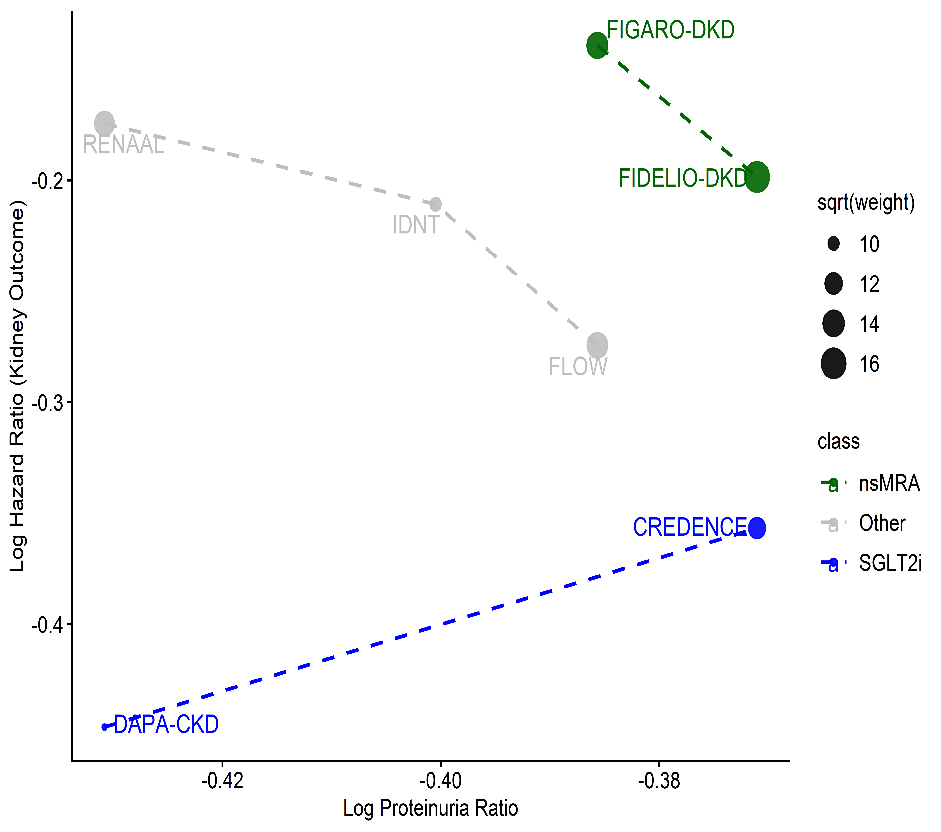
*

**Figure S13. Trial-Level Association between Albuminuria Reduction and Kidney Outcomes Stratified by Class**

Scatter plot of trial-level associations stratified by drug class, including sodium glucose co-transporter 2 inhibitor (SGLT2i) in blue, nonsteroidal mineralocorticoid receptor antagonists (nsMRA) in green, and other therapies trials (grey). Bubble size reflects study weight. Dashed lines connect trials within each class to illustrate within-class directional patterns. No regression modeling was performed within subgroups. Visual inspection suggests a consistent directional relationship among SGLT2i trials, whereas nsMRA trials and other class trials demonstrate heterogeneous patterns, suggesting potential mechanism-specific differences in the relationship between albuminuria reduction and clinical outcomes.

*Table S6. Comparison with Prior Network Meta-Analysis*

| Characteristic | Previous published NMAs | Present study |
| --- | --- | --- |
| Literature search | Through 2023 | Through April 2026 |
| Included landmark trials | CREDENCE, DAPA-CKD, EMPA-KIDNEY, FIDELIO-DKD, FIGARO-DKD, SONAR, IDNT, RENAAL | All previous trials plus FLOW |
| GLP-1RA kidney outcome data | Limited | Dedicated kidney outcome trial (FLOW) included |
| CINeMA assessment | No | Yes |
| Formal transitivity assessment | Limited | Prespecified effect modifiers summarized |
| Interpretation of rankings | Comparative rankings reported | Rankings deliberately omitted |
| Primary interpretation | Comparative efficacy | Placebo-referenced class-level treatment effects |
| Principal conclusion | Suggested relative treatment differences | Emphasized limitations of indirect comparisons and avoided comparative superiority claims |

* Comparison of the present network meta-analysis with prior published network meta-analyses, highlighting differences in included evidence, methodological approach, and interpretation of findings.
